# Advances in targeted therapy for tumor with nanocarriers: A review

**DOI:** 10.1016/j.mtbio.2025.101583

**Published:** 2025-02-15

**Authors:** Hongxia Cheng, Juan Liao, Yuhan Ma, Muhammad Tariq Sarwar, Huaming Yang

**Affiliations:** a Engineering Research Center of Nano-Geomaterials of Ministry of Education, China University of Geosciences, Wuhan, 430074, China; b Laboratory of Advanced Mineral Materials, China University of Geosciences, Wuhan, 430074, China; c Faculty of Materials Science and Chemistry, China University of Geosciences, Wuhan, 430074, China; d Hunan Key Laboratory of Mineral Materials and Application, School of Minerals Processing and Bioengineering, Central South University, Changsha, 410083, China

**Keywords:** Cancer therapy, Nanocarriers, Targeted therapy, Nanoclay, Regulation

## Abstract

The application of Nanocarriers (NCs) provides a promising strategy to solve the problems faced by traditional chemotherapy drugs, like the imprecise delivery, poor bioavailability, high dose requirement, and the tendency to develop multidrug resistance. With the protection of NCs, chemotherapy drugs can reach the lesion site and then release accurately and completely. Although some reviews have summarized the biological applications of NCs, little attention has been given to the advantages and disadvantages of analyzing organic, inorganic, and hybrid NCs separately for targeted therapy and identifying means to further improve the targeting ability. First, in this review, we emphasize three factors that have a marked impact on targeted therapy: the tumor microenvironment (TME), the different administration modalities (intravenous, oral, and intracavitary administration), and the targeting pathways (passive and active). Second, a systematic examination of the advantages and disadvantages of polymeric NCs, dendrimers, micelles, liposomes, mesoporous silica NCs, gold NCs, quantum dots, nano clay, core-shell NCs, and MOFs for targeted therapy are reviewed. Further, we propose three ways to improve the efficiency of targeted therapy, including regulating the size, shape, and surface properties of NCs.

## Graphical abstract

**Figure:**
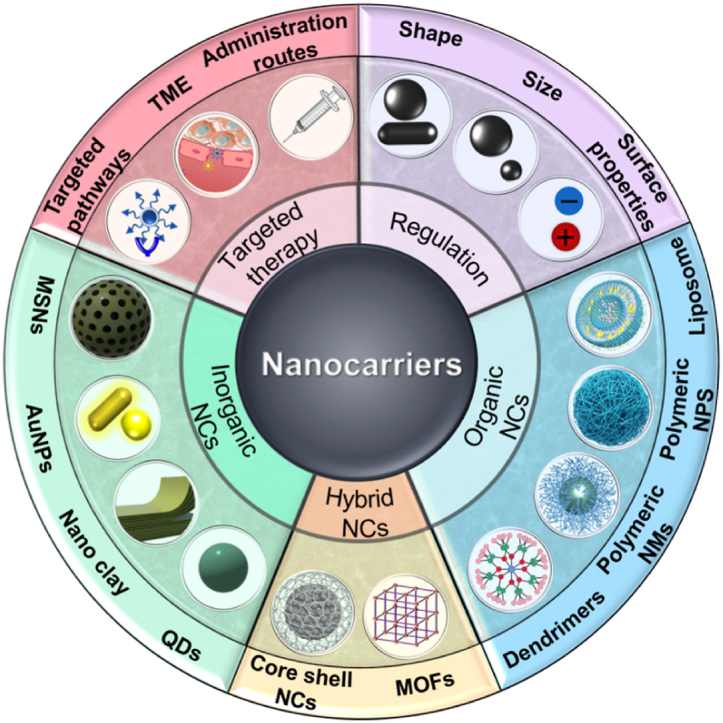

## 1 Introduction

Tumor is a disease formed by abnormal cell proliferation, with the most prominent feature being mass formation in the body. Finding new drug delivery technology is an urgent task to improve the efficiency of tumor therapy [1]. Although a variety of treatments (such as surgical resection, chemotherapy, and radiotherapy) have been created to enhance clinical cancer treatment, the effects of treatment on tumor recurrence and metastasis are still unsatisfactory [2]. When a patient has an invasive malignant tumor or undergoes a sentinel lymph node biopsy (SLNB), local treatment may not suffice, and chemotherapy can be more effective [3]. However, the toxicity and side effects, poor solubility, poor stability, and limited specificity of most chemotherapy drugs seriously limit the prospects for continued application and favorable outcomes of conventional chemotherapy drugs. With the continuous advancement in the precision of diagnosis and specificity of treatment, nanocarriers (NCs) have emerged as a promising modality in medicine, assisting in the delivery of traditional chemotherapy drugs to overcome limitations in clinical practice or for research studies. Numerous studies have shown that they have the potential to improve anticancer efficacy and reduce drug side effects [[4], [5], [6]].

Delivering drugs to specific sites of lesions is one of the major challenges of chemotherapy [7]. NCs that can comprehensively control the distribution of drugs *in vivo* in space, time, and dose have become a research hotspot [8]. NCs can be loaded with one or more drugs and delivered to tumors through active or passive targeting mechanisms. These pathways enable drugs to accumulate in tumor tissues and reduce the harmful effects of the drugs [9]. However, due to the different delivery challenges and complex tumor microenvironments (TME) faced by each drug form or technology, it is difficult to overcome all obstacles in the design of NCs, making translation of NCs into clinical practice very difficult [10].

Ideal NCs need to achieve precise drug delivery and local disease control with minimal systemic toxicity [11]. Maeda and colleagues discovered the enhanced permeability and retention (EPR) phenomenon, which enables the retention and accumulation of NCs at tumor sites [12,13]. This has provided an opportunity for the precise delivery of chemotherapy drugs and makes the delivery of NCs to cancer cells complex [14]. However, targeted delivery *in vivo* is far more complicated than initially anticipated. NCs must evade immune surveillance and the binding of serum regulatory proteins. Achieving homogeneous distribution within the tumor necessitates surmounting multiple obstacles. For instance, intratumoral injection demands advanced imaging-guided techniques and highly skilled operators to minimize injection errors and prevent damage to surrounding normal tissues. The thickness of the tumor stroma, acting as a physical barrier, restricts the penetration and spread of injected substances, thus requiring the development of strategies to modify the stromal architecture or enhance the agent's ability to traverse this obstacle. Additionally, macrophage uptake can sequester a significant portion of the injected agent, reducing its availability for tumor targeting. Understanding the mechanisms of macrophage-agent interaction and devising methods to evade or modulate this process is of utmost importance. The elevated interstitial fluid pressure within the tumor hinders the dispersion of the agent, warranting innovative approaches to relieve this pressure or designing agents that can function effectively under such conditions. The inherently slow diffusion rate of substances in tumor tissue necessitates the exploring methods to accelerate the spreading process, such as employing carriers with enhanced diffusivity or applying external physical forces. By systematically addressing these challenges, the goal of achieving uniform distribution throughout the tumor and improving therapeutic efficacy can be realized [15].

**Fig. 1:**
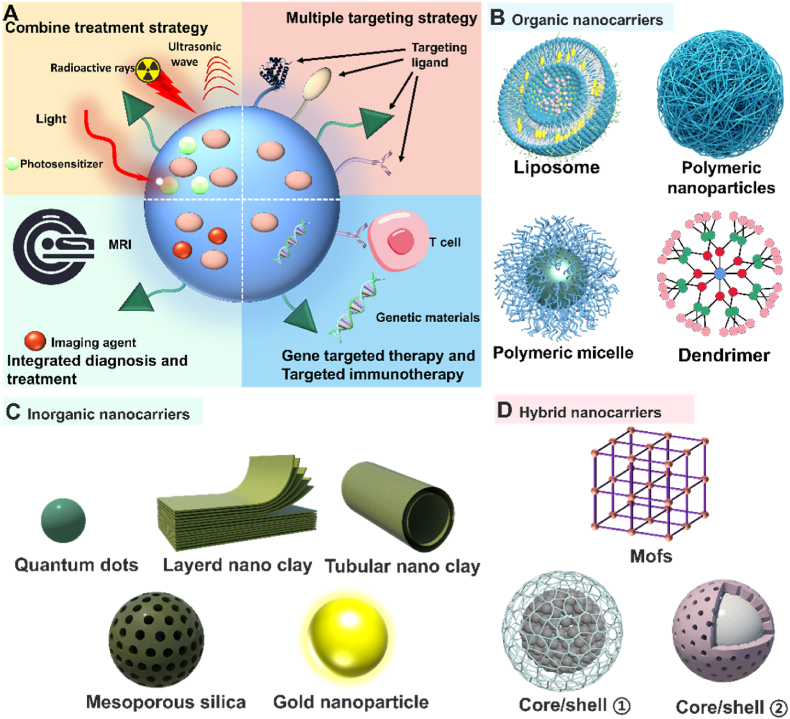
Different types of nanocarriers (NCs) for targeted therapy. (A) NCs for different targeted combination therapy strategies and functionalization; (B) Organic nanocarriers; (C) Inorganic nanocarriers; (D) Hybrid nanocarriers.

The size, surface properties, and chemical composition of NCs, among other physical or chemical properties, can be regulated [16]. NCs can enhance drug delivery efficiency by altering the pharmacokinetics and pharmacodynamics of drugs, and they can also achieve diverse functions. These functions include targeted therapy through surface ligand grafting, diagnosis, and treatment via contrast agent grafting, photothermal targeted combination therapy by adding photosensitizers or using materials with high photothermal conversion, and controlling drug release location and duration by coating sensitive response materials (Fig. 1A) [[17], [18], [19], [20], [21]].

This review divides NCs into three categories: organic NCs (Fig. 1B), inorganic NCs (Fig. 1C), and hybrid NCs (Fig. 1D). The characteristics of each NCs and the latest scientific research progress were collected and summarized. The development of targeted therapy for cancer is inseparable from the need to advance the nanotechnology of drug carriers [22,23]. In this review, we pay attention to the pathways of NCs to achieve targeted positions, some paradigms that respond to TME, and some ideas for regulating NCs to improve the efficiency of targeted therapy. At the same time, some interesting designs of NCs in recent years are presented, and then the factors affecting the properties of NCs are analyzed. Finally, we put forward our opinions and predictions on the future development trend of NCs.

## 2 Targeted therapy

Targeted therapy is a treatment method that targets specific oncogenic sites (protein molecules or gene fragments within tumor cells) at the cellular and molecular levels [24]. This therapy enables personalized and precise treatment of different pathologies. With the help of NCs, it is possible to further multifunctional therapeutic molecules, improve the diagnosis and treatment efficiency of therapeutic molecules through targeted therapy, improve the phenomenon of burst release, control the amount and location of drug release, and thus reduce toxicity. An in-depth study of the characteristics of each TME is the basis for us to choose the methods of administration and targeting pathways of NCs [25]. NCs can be administered orally [26], intravenously [27], or intracavitary [28], allowing for either systemic or local distribution. Currently, there are mainly two types of targeting pathways for NCs: one is passive accumulation in tumor tissue relying on the EPR effect, while the other is the active accumulation of ligands that can specifically bind to overexpressed receptors, transporters, and integrins on the surface of tumor cells in tumor tissue [29,30].

### 2.1 Tumor microenvironment

**Fig. 2:**
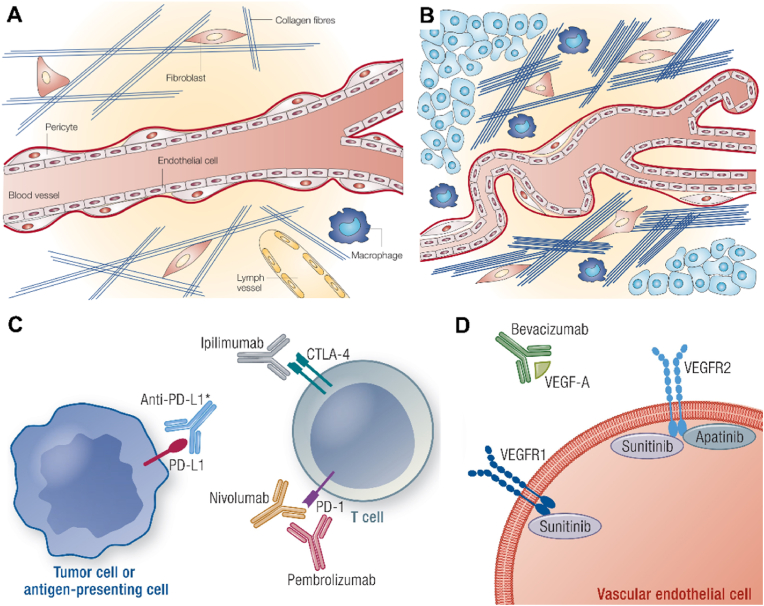
The difference in composition between normal tissue and tumor tissue. (A) The normal tissue microenvironment. Normal tissue blood vessels are smooth and complete, with smooth endothelial cells and pericutaneous cells scattered along the capillary wall interval. The extracellular matrix is composed of fibroblasts, macrophages, a sparse network of collagen and other fibers, and some lymphatic blood vessels. (B) The tumor microenvironment. Vascular abnormalities in tumor tissues include uneven blood vessel form and leakage, a significant number of vesicles, and excessive endothelial cell activity. Tumor tissue blood arteries have fewer pericytes, resulting in decreased vascular stability. Furthermore, many tumors lack lymphatic veins, making efficient clearance of interstitial fluid and soluble proteins problematic. Tumors have a dense network of collagen fibers thicker than normal tissue in their extracellular matrix. A massive number of fibroblasts are also present in the tumor, which attach to the collagen fibers and enhance the tension between them. There is an increase in the number of macrophages and other inflammatory cells. (A–B) Reproduced with permission [33]. Copyright 2004, Royal Society of Chemistry. (C) Mechanism of immune checkpoint inhibitors (ICIs). (D) Anti-VEGF-A and anti-VEGFRs in the treatment of hypophysis and aggressive pituitary adenomas. (C–D) Reproduced with permission [43]. Copyright 2022, Oxford University Press.

Targeted drug delivery that tracks down the TME is thought to hold great promise because the TME not only plays significant roles in tumor progression and metastasis but also has a significant impact on the outcome of tumors [31]. TME composition varies among different tumor types but mainly includes blood vessels, stromal cells, immune cells, and extracellular matrix [32]. Significant structural differences exist between normal and tumor tissue (Fig. 2 A&B) [33]. Normal tissue is spreading and unbroken, consisting of a smooth layer of endothelial cells and pericytes located at intervals along capillary walls [34]. A sparse collagen and other fibers network and a few fibroblasts, macrophages, and lymphatic capillaries contribute to the extracellular matrix. In contrast, tumor tissue is leaky with vascular defects, irregular shape, numerous vesicles, and highly activated endothelial cells [35]. As a result, blood circulation is inadequate to oxygenate tumor tissue, often making it hypoxic [36]. These vessels have fewer pericytes than normal tissues, resulting in reduced vascular stability and proliferative potential [37]. In addition, many tumors lack lymphatic vessels, making it difficult to effectively clear interstitial fluid and soluble proteins [38].

The extracellular matrix of tumors features a dense collagen fiber network, endowing tumor cells with rigidity. Concurrently, the abnormal metabolism of tumor cells, characterized by a high demand for oxygen and the substantial release of proteases, fibroblasts, and macrophages, gives rise to a tumor immunosuppressive microenvironment. One suspects, therefore, that an in-depth study of the characteristics of the TME, using the basic physiopathological features of tumors and analyzing their role in tumors, would improve the efficiency of targeted therapy and help us treat cancer [9]. Currently, NCs responsive to reactive oxygen species, hypoxia, and low pH for TME have become a popular strategy for targeted therapy [39].

TME possesses a highly intricate composition and undergoes continuous evolution as the tumor progresses [40]. TME has both a positive and negative impact on the outcome of tumor treatment, depending on the type of tumor, its stage of development, the developmental impact of individual TME cells, and the organ in which they reside [41]. At the same time, TME plays a vital role in responding to therapeutic interference, manifesting as intrinsic or acquired/adaptive resistance [42]. Intrinsic resistance of the TME refers to the nature of resistance to invading substances, which is mediated by the environment, including elevated interstitial fluid pressure (IFP) and low vascular supply efficiency, both of which prevent the accumulation and distribution of drugs at the tumor site, so that tumor cells are temporarily protected [43]. Large numbers of immunosuppressive cells promote intrinsic resistance [44]. Acquired drug resistance is defined as the adaptive response to therapeutic interference of the host [45]. Acquired resistance develops over time due to genetic changes in the sequence that ultimately lead to a complex therapeutic resistance phenotype [46]. Many studies have reported pleiotropic adaptations to TME components and phenotypes following various therapeutic interventions [47]. Recognizing the importance of TME broadens our thinking about new possibilities for implementing cancer therapy [48,49]. For example, knowledge of the relationship between non-tumor components of the TME or non-tumor components and tumor components helps us treat cancer [50]. Each component of the TME is complex and represents a potential target for cancer treatment [51].

Pituitary adenomas (PAs) are the second most common brain tumor, accounting for 10–25 % of all intracranial tumors [52]. They originate from highly differentiated anterior pituitary cells, which release one or more hormones [37]. The majority of PAs develop slowly and are common benign tumors, and they may be readily treated with existing medical therapies, surgery, and radiation [53]. However, a minority proportion of PAs demonstrate malignant biological characteristics, such as aggressive fast growth and metastasis, and do not respond to standard surgery, radiation, or pharmacological therapy [54]. Their biology, and their diverse spectrum of behavior are only partially known [55]. For instance, there are no medicinal treatments for gonadotroph adenomas (non-functional pituitary adenomas) [43]. Treating severe PAs and pituitary carcinomas can also be challenging. In-depth research into the microenvironment of invasive PAs and the search for therapies targeting TME can lead us to better treatments [24]. Immune checkpoints (IC), defined as programmed death receptors and their ligands, are a group of molecules produced on immune cells that control the level of immunological activity [56]. Tumor cells will express certain chemicals to activate IC, and once activated, IC acts as a "brake," preventing antigen from being given to T cells and thereby inhibiting the antigen presentation process in the tumor immunological link [57]. This reduces T cell immune activity, allowing the tumor to avoid detection and survive. Immunological checkpoint inhibitor (ICI) treatment is a novel type of immunotherapy that enhances the immunological milieu around the tumor to reawaken immune cells in the body for anti-tumor purposes [58]. In the microenvironment of PAs, ICIs can block immune checkpoints to prevent their inhibition and negative regulation of immune cells, including cytotoxic T-lymphocyte-associated protein-4 (CTLA-4) and programmed cell death protein-1 (PD-1) on T cells or programmed cell death protein ligand-1 (PD-L1) on antigen-presenting cells and tumor cells (Fig. 2C) [59]. Antiangiogenic therapy is a kind of targeted therapy, which can reduce the blood supply to the tumor growth site through drugs, block the nutrient supply of the tumor, and inhibit the tumor growth, so as to treat the tumor more effectively. To date, clinical anti-angiogenic therapies for invasive PAs include bevacizumab, an anti-VEGF-A monoclonal antibody, and anti-tyrosine kinase inhibitors Sunitinib (which primarily targets VEGFR1 and VEGFR2) and Apatinib (which primarily targets VEGFR2) (Fig. 2D).

TME research in the field of cancer treatment has great potential, and NCs play an important role in it. Precise and personalized treatment depends on an in-depth understanding of TME. NCs can achieve passive targeting by virtue of their size advantages. Their nanoscale allows them to take advantage of the vascular characteristics of tumor tissue, such as its high permeability and inefficient lymphatic drainage system, to enrich drugs at the tumor site. Through surface modification with specific ligands, active targeting can also be achieved, enabling them to accurately identify and bind to tumor cells. Moreover, intelligent responsive NCs can control drug release using TME signals (such as pH, enzyme concentration). For example, in an acidic tumor microenvironment (lower pH), certain NCs can be designed to change their structure or release the encapsulated drug. This mechanism helps reduce toxic side effects by ensuring that the drug is released precisely where needed. Nevertheless, the application of NCs is fraught with numerous challenges. Individual differences significantly affect the TME, necessitating that NCs be customized according to the characteristics of TME of different patients to achieve individualized treatment. For instance, different patients may exhibit varying expression levels of tumor cell surface receptors, distinct pH ranges in TME, and diverse enzyme activities. NCs must be able to adapt to these variations. Future research could center on developing novel surface modification techniques. For example, nanobiomimetic coating technology can precisely modify the surface properties of NCs to enhance their affinity for specific tumor cell surface receptors. Additionally, advanced imaging technologies can be exploited to better understand patient-specific TME. This will enable the design of more personalized NC systems, ensuring accurate drug delivery and effective release. Furthermore, further studies are required to optimize the intelligent response mechanisms of NCs. More clinical trials and preclinical data are essential to validate their effectiveness in reducing toxic side effects.

### 2.2 Administration route

An ideal drug delivery system achieves rapid absorption, long-duration drug action, and minimal side effects, all of which depend on the route of administration [60]. The function of NCs in this effort is to enable the slow release of drugs over a long period while maintaining a constant and controlled drug concentration in the blood or within target tissues [61]. Oral, intravenous, and intracavitary delivery are the most common modes of delivery of anti-cancer drugs [37]. Selecting suitable, functional materials to construct diversified NC systems to overcome the obstacles of biological drug delivery remains a critical problem that needs to be solved [62].

#### 2.2.1 Oral administration

Oral administration is preferred due to its non-invasive nature, which avoids the discomfort caused by injection and reduces the likelihood of infection [63]. It shows the highest level of patient compliance and is amenable to self-administration, reducing the physical and psychological adverse effects [38].

**Fig. 3:**
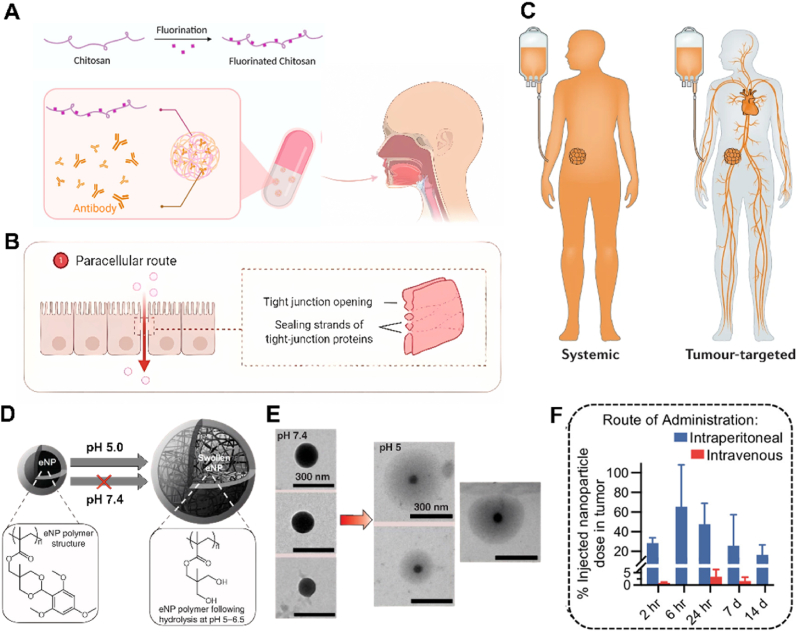
Different modes of administration are used for tumor targeted therapy. (A) Fluorocarbon modified chitosan (FCS) and its self-assembly with the immune checkpoint antibody to form a nanocomplex, then freeze dried and encapsulated in enteric-coated capsules as an oral drug. (B) The illustration of the paracellular delivery pathway using tight junction openings.(A–B) Reproduced with permission [57]. Copyright 2023, American Chemical Society. (C) Comparison of immunotherapy delivery strategies graphically showing the typical biological distribution of systemic and tumor-targeted immunotherapies administered immunotherapies. Reproduced with permission [199]. Copyright 2021, Springer Nature. (D) Structural changes of eNPs in different pH environments. (E) TEM of eNPs maintained at pH 7.4 (left) or pH 5 (right). (F) Nanoparticle content in tumors at different periods after intravenous or intraperitoneal administration. (D–F) Reproduced with permission [28]. Copyright 2023, American Chemical Society.

However, the bioavailability of orally administered drugs is often not ideal, mainly due to the five major obstacles that oral drug preparations face once they enter the human body [57]. The first obstacle is the pH gradient in the gastrointestinal tract (GIT) [58]. Take oral colon cancer drugs as examples; after entering the body, the drug will first pass through the acidic environment of the stomach at pH 1–3 and stay for 0–6 h, then enter the duodenum at pH 6 and the end of the ileum at pH 7.4, which takes about 2–6 h, finally reach the colon at pH 6–8 [46]. And yet, precisely due to this sharp change in pH, it provides us with ideas for designing targeted NCs [58]. The variable pH can be harnessed to develop pH-responsive NCs, ensuring the drug's safe transit to the lesion site and enabling its release [46]. Meanwhile, the development of pH-responsive targeted drug delivery systems must consider the pathophysiology of the GIT lesion, as the pH value of the tissue will generally decrease after the cancer. The second obstacle is a large number of enzymes in the GIT, such as pepsin, trypsin, and glucosidase, which are also non-negligible hindrance factors [64]. After oral administration, drugs may be digested or degraded by these enzymes in the GIT and small intestine, and it is difficult to reach the lesion tumor site completely [57]. Therefore, some carriers with strong stability, such as clay minerals and polysaccharides, can be selected to protect drugs from degradation [65]. The third barrier is the mucus barrier, which consists primarily of crosslinked mucin fibers ranging in size from 0.5 to 40 MDa but also contains other proteins, carbohydrates, lipids, bacteria, and cell debris [57]. In addition, mucus behaves as a viscoelastic fluid. These properties can affect drug absorption. Studies have shown that drug-carrying nanoparticles of 100 nm and smaller are more likely to pass through the mucus barrier. Bionic methods can also avoid mucus clearance, such as coating red blood cell membranes on the drug surface [66]. In addition, pegylation can also be modified on the surface of the NCs to reduce the immunogenicity of the NCs and improve stability and cycle time, which has been used in clinical applications for more than 30 years [67]. The fourth obstacle is the epithelium. The epithelial cells of the gut are designed to prevent the transfer of toxins and gut bacteria into the blood circulation. Intestinal epithelial cells are tightly stacked and connected by tight junction proteins. Such tight connections between epithelial cells make it hard for drugs to penetrate and even harder for macromolecules [68]. Oral drugs are mainly transported through epithelial cells through paracellular or transcellular transport. Transcellular transport is carried out by cells and is usually controlled by transmembrane proteins and several cytoplasmic proteins [63]. Paracellular transport includes passive diffusion, active transport, and endocytosis [35]. Endocytosis is the most common route for drug molecules to cross the small intestine. Paracellular transport channels account for only 0.01–0.1 % of the total intestinal surface area. In addition, drugs with strong hydrophilicity are difficult to absorb effectively through the paracellular pathway. Paracellular transport is also limited by the size of drug molecules, with small molecules in the 100–200Da range relatively easy to absorb. In addition, the use of osmotropic agents can increase the ability of the drug to penetrate the intestinal epithelium. Oral protein drugs, especially large proteins (such as antibodies), are a massive challenge because they have difficulty penetrating the intestinal barrier. Zhu et al. [57] mixed fluorocarbon-modified chitosan (FCS) with therapeutic proteins to form nanoparticles and obtained an antibody-class drug with a bioavailability of 4.73 % (Fig. 3A). In their study, it was found that FCS was able to induce a brief rearrangement of proteins associated with tight junctions between epithelial cells to promote mucosal intestinal intercellular protein cargos transmission through the paracellular pathway (Fig. 3B). In addition to the paracellular pathway, FCS has also been found to encourage the endocytosis pathway, which facilitates the transmucosal transfer of proteins. During osmosis, FCS/protein nanocomplexes can be dissociated, and FCS interact with epithelial cells to promote the infiltration of protein carriers and free proteins into the blood circulation. In addition to the four obstacles mentioned above, the microbial flora in the GIT can also affect drug absorption. There are hundreds of kinds of microbes in the GIT that are involved in the digestion and metabolism of fatty acids, carbohydrates, and proteins. In addition, microbes in the GIT can directly metabolize cholesterol in the diet, thus affecting the solubility and permeability of drugs. These five major obstacles have seriously hindered the development of oral NCs, and are also the key points to be overcome by oral NCs.

Although the targeted delivery mechanism of oral drug NCs is constantly being optimized, integrating multiple targeting mechanisms has brought significant technical complexity. For example, when combining pH response with enzyme response and other targeted mechanisms, accurately calibrating their combined effects during the production process becomes a challenging task, which often leads to inconsistent performance of NCs, and some may release drugs too early or too late, impairing the overall therapeutic effect. Moreover, individual differences have posed significant obstacles to the successful application of oral drug NCs. There are substantial differences in the physiological characteristics of the gastrointestinal tract among different patients. In terms of gastric acid secretion, some patients with higher levels of gastric acid may accelerate the decomposition of NCs before they reach the expected drug release site, while others with lower levels of gastric acid may hinder the timely dissolution and release of drug payloads; The fluctuation of intestinal peristalsis speed can alter the delivery time of NCs, thereby changing the optimal drug release site and reducing absorption efficiency; In addition, the composition of gut microbiota varies among individuals, and its interaction with NCs is difficult to predict. Some microbial species may enzymatically hydrolyze the components of NCs or alter the local environment in a way that affects drug bioavailability. The combined factors of technological complexity and individual differences pose significant obstacles to the clinical translation of oral drug NCs, severely limiting their widespread application. Although recent advances in this field have shown great potential, to fully realize its clinical benefits, it is necessary to overcome various difficulties in technology, practical application, cost, and clinical translation. This requires joint efforts from research institutions, medical professionals, and industry sectors to continuously improve and perfect this technology.

#### 2.2.2 Intravenous administration

Intravenous injection, as the main route of administration for most current anti-tumor drugs, does have certain advantages [60]. Direct injection of drugs into the bloodstream can quickly reach the pathological site, and the predictability of serum pharmacokinetics ensures good drug efficacy, which is crucial for timely control of disease progression in tumor treatment [69]. However, its disadvantages cannot be ignored. From the perspective of patient experience, the pain and discomfort caused by invasive procedures directly affect the quality of life of patients, especially for those who require long-term repeated treatment; the cumulative effect of this pain is more significant. The increased risk of infection not only increases the physical burden on patients but may also prolong hospitalization time, increase medical costs, and even cause other serious complications due to infection, endangering the patient's life. Endovascular inflammation and superficial thrombophlebitis limit patients' mobility and impair their daily activities and rehabilitation process, impairing vascular function and resulting in long-term adverse effects on overall health [70].

In terms of therapeutic efficiency, although NCs have particular potential, they are quickly cleared by the reticuloendothelial system after intravenous injection, which significantly hinders their full potential and prevents drugs from exerting sustained and effective therapeutic effects at the lesion site, resulting in drug waste and possibly promoting tumor cells to develop drug resistance. Meanwhile, although the systemic toxicity associated with intravenous injection can be partially alleviated through targeted therapy, the effectiveness of targeted therapy still needs to be further improved, and there are individual differences(Fig. 3C). Not all patients can benefit ideally from it. In addition, potential adverse reactions such as autonomic nervous system, skin and mucous membrane, and cardiopulmonary reactions caused by intravenous injection, although the mechanism is unclear, undoubtedly bring additional health risks to patients, and may manifest differently due to individual differences, making it difficult to predict and effectively prevent in advance. These unclear risk factors increase uncertainty in clinical applications and highlight the need for in-depth research on their mechanisms [61]. Overall, intravenous administration is valuable in anti-tumor therapy, but there is an urgent need for improvement and optimization to enhance patient treatment tolerance, reduce risks, and improve treatment efficacy. At the same time, further research should be conducted on the mechanisms of adverse reactions related to intravenous injection to provide a more scientific basis for clinical decision-making and promote the development of tumor treatment in a safer and more efficient direction.

#### 2.2.3 Intracavitary administration

Intracavitary administration, such as thoracic and peritoneal administration, provides direct access to the tumor cavity and thus offers the chance to deliver higher concentrations of the payload drug. Higher drug load often exacerbates inflammatory response in these cavities, such as producing abdominal adhesion that leads to intestinal obstruction and abdominal pain. Intracavitary delivery provides intrinsic proximity and direct surface contact of the drug with the tumor. Due to this proximity advantage, it is commonly used in patients when conventional chemotherapeutic agents, such as paclitaxel or cisplatin, are delivered to the mucosal lining and surface of vital organs within the cavity.

Expandable nanoparticles (eNPs) are a class of nanoparticles that can change their size in response to environmental changes. Colby et al. [28] reported an eNPs capable of swelling in the TME (pH 6.5) or the late-onset endosome/lysosome of a single tumor cell (pH 5), and the eNPs particle size increased from 100 nm to 1000 nm at low pH (Fig. 3D and E). This targeted strategy based on material properties relies on pH changes in the TME and is highly tumor-specific. They found that the overall delivery efficiency of eNPs to tumor tissue by intraperitoneal injection was 30 % of the injected dose after 1–2 weeks. This drug delivery efficiency is much higher than other drug delivery methods (Fig. 3F), and it has an apparent targeting ability. This illustrates the impact of nanoparticle delivery methods on targeting efficiency and therapeutic efficiency and encourages us to explore more advantageous delivery routes to improve the efficiency of tumor therapy with as few side effects as possible to improve its clinical transformation.

Intracavitary administration has unique advantages in increasing local drug concentration compared to oral and intravenous administration. However, it also faces challenges like managing the inflammatory response. The inflammatory response may cause increased patient pain and affect organ function because of tissue adhesion. Future research should focus on optimizing pharmaceutical formulations and dosages for intracavitary administration to balance therapeutic efficacy and adverse reactions. For example, current research faces difficulties in material selection and preparation processes in developing intracavitary drug delivery formulations with sustained release function. Nevertheless, such formulations are expected to maintain a high concentration of local drug action while reducing the stimulation of surrounding tissues by sudden drug release. At the same time, the combination of anti-inflammatory drugs and chemotherapy drugs for intracavitary administration strategy is also a promising direction. For instance, the combination of non-steroidal anti-inflammatory drugs with specific chemotherapy agents may enhance the anti-tumor effect by reducing inflammation-induced immunosuppression and directly targeting cancer cells. This strategy is expected to improve the efficacy of tumor treatment while reducing inflammation-related complications. By addressing these aspects, the potential of intracavitary administration in cancer treatment can be further maximized.

### 2.3 Targeted pathways

**Fig. 4:**
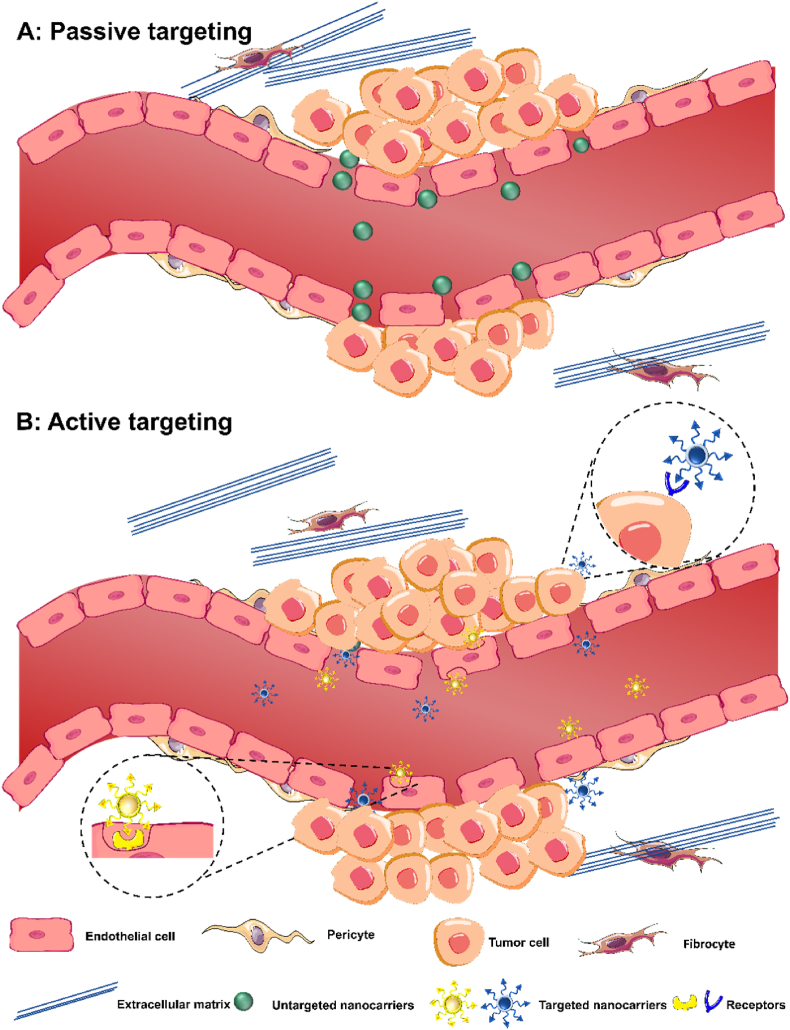
Pathways to target tumor cells. (A) Passive targeting strategies. NCs rely on the EPR effect to penetrate tumor tissues from irregular vascular spaces in TME and remain in tumor tissues. (B) Ligands on the surface of the NCs bind specifically to receptors overexpressed by tumor cells or endothelial cells and rely on receptor-mediated endocytosis on the cell surface to enter the tumor tissue or cells and kill the tumor.

The targeting pathways of NCs are generally divided into passive targeting (Fig. 4A), and active targeting (Fig. 4B). The characteristics of tumor cells and the physicochemical properties of NCs play a decisive role in selecting targeting strategies [71]. A passive targeting strategy requires encapsulation in a carrier with a high blood half-life [72]. Active targeting strategies are preferred for drugs that are difficult to transit across membranes and may cause severe damage to normal cells [73]. However, active targeting requires surface modification with ligand decorations, often reflective of a complex process [74]. The active targeting technique has several challenges due to the complexity of the human body environment. After entering the bloodstream, the NCs will come into contact with and interact with biological macromolecules (such as carbohydrates, proteins, nucleic acids, and lipids), which can cover the NCs, causing surface or biomolecular corona to change the surface properties of the NCs and impair targeting ability [75]. Therefore, the implementation and clinical translation of active targeting strategies are very difficult and in urgent need of a breakthrough.

#### 2.3.1 Passive targeting

Passive targeting therapeutic strategies rely heavily on EPR effects that describe the distribution and fate of delivered nanoparticles on major tumors (>1 cm^3^) when associated with a circulatory system that is very porous or "leaky" [76]. Most current target treatments are passive [77]. Under EPR effects, the vascular endothelium is more permeable than in a healthy state, and hypoxia with mild acidity is characteristic of the TME. Due to their rapid expansion, tumors need to form new blood vessels to get nutrients and oxygen to ensure that the tumor grows exponentially. The tumor mesenchyme is selectively breached by improved penetration of macromolecules and NCs larger than 40 kDa, through the recently generated leaky capillaries. Consequently, nanoscale drug carriers tend to accumulate in the tumor and target site due to their reduced propensity to diffuse into healthy normal tissue, attributed to the enhanced permeability and retention (EPR) effect and specific interactions with tumor cells.

Many encouraging results have been seen using this therapeutic approach, including enhanced concentration of the medications at the target sites and less toxicity in healthy tissue. Numerous passive targeting NCs, including liposomes, micelles, albumin-coated nanoparticles, and polymeric nanoparticles, have been authorized for use in the treatment of various tumors over the past 20 years. However, reliance solely on the EPR effect limits the possible selectivity. Notably, recent studies have questioned the entry mechanism of NCs into solid tumors. Sindhwani et al. [78] explored NCs tumor penetration, finding evidence that transcytosis may be the main mechanism of NCs enrichment at tumor sites. The endothelial cell space, long considered a key factor in the EPR effect, was found to be less frequent. Among 313 blood vessels in all tumor models, only 26 gaps were detected, with a total gap coverage of 0.048 % of the vessel surface area and the number of interendothelial spaces 60 times less than the accumulated NCs. This partially supports the EPR effect but fails to fully explain the accumulation of NCs. In the past 30 years, NCs have multiplied and become more complex. Table 1 lists the approved nanocarrier-based drugs of the Food and Drug Administration (FDA) and the European Medicines Agency (EMA). As chemotherapy NCs, they reduce systemic toxicity and improve patient tolerance. However, the high failure rate during clinical trials has led to doubts about their efficacy. The poor performance of NCs mainly results from insufficient tumor accumulation and unfavorable pharmacokinetics. These findings not only challenge the long-held assumptions about the EPR effect but also prompt a reevaluation of the current design strategies of NCs. The limited understanding of tumor spillover mechanisms is a significant obstacle that needs to be overcome. It is essential to shift the focus from merely relying on the EPR effect to a more comprehensive consideration of multiple NCs parameters. For instance, further investigations into how material composition, geometry, surface chemistry, charge, and mechanical properties interact with biological barriers could pave the way for developing more efficient and effective NCs. This would potentially revolutionize the field of cancer nanotherapy and offer new hope for patients suffering from malignancies.

**Table 1:** Summary of NCS-based drugs which have been approved by the FDA or EMA.

Drug Name	Approval Year	Applicable Cancer Types	Ref.
Doxil® (Doxorubicin Liposome)	1995 (FDA), 1996 (EMA)	AIDS-related Kaposi's sarcoma, breast cancer, ovarian cancer, etc.	[201]
DaunoXome® (Daunorubicin Liposome)	1996 (FDA)	Kaposi's sarcoma	[202]
Oncaspar (Polyethylene glycol-conjugated asparaginase)	1994 (FDA), 2006 (EMA)	Acute lymphoblastic leukemia	[87]
DepoCyt® (Cytarabine Liposome)	1999 (FDA)	Lymphomatous meningitis	[203]
Myocet® (Doxorubicin Liposome)	2000 (EMA)	Metastatic breast cancer	[204]
Abraxane® (Albumin-bound Paclitaxel)	2005 (FDA), 2008 (EMA)	Advanced non-small cell lung cancer, metastatic breast cancer, metastatic pancreatic cancer, etc.	[88]
Mepact® (Mifamurtide Liposome)	2009 (EMA)	Osteosarcoma (first-line treatment after surgery)	[205]
Nanotherm® (Magnetic iron oxide nanoparticles)	2010 (EMA), 2013 (FDA)	Recurrent glioblastoma, prostate cancer, and pancreatic cancer (thermal ablation treatment)	[113]
Marqibo® (Vincristine Liposome)	2012 (FDA)	Philadelphia chromosome-negative acute lymphoblastic leukemia (third-line treatment)	[206]
Onivyde® (Irinotecan Liposome)	2015 (FDA), 2016 (EMA)	Metastatic pancreatic cancer	[207]
Vyxeos® (Cytarabine/Daunorubicin Liposome Complex)	2017 (FDA), 2018 (EMA)	Acute myeloid leukemia	[208]
Apealea® (Paclitaxel Polymer Micelle)	2018 (EMA)	Non-small cell lung cancer, ovarian cancer, and breast cancer	[89]
Hensify® (Hafnium Oxide Nanoparticle)	2019 (EMA)	Locally advanced soft tissue sarcoma (adjuvant radiotherapy)	[209]
Pazenir ratiopharm (Albumin-bound Paclitaxel)	2019 (EMA)	Metastatic breast cancer, metastatic pancreatic cancer, non-small cell lung cancer	[210]
Fyarro® (Sirolimus albumin binding granules)	2021 (FDA)	Epithelioid cell tumor	[211]

#### 2.3.2 Active targeting

Active targeting uses specific ligands that can specifically bind to one or more receptors overexpressed on tumor cells to accumulate drugs at designed sites (Table 2) [79]. This specific ligand requires having a smaller affinity for normal cells and a stronger affinity for tumor cells to enhance the biosafety of the NCs, which usually depends on the proportion of specific receptors expressed between tumor cells and normal cells [80]. The ideal targeting ligand requires three characteristics [80]. The first characteristic is to select receptors expressed on the cell surface rather than inside the cell because the membrane permeability of ligands after grafting NCs is considerably reduced, making it difficult to enter the cell interior. At the same time, once the NCs have good membrane permeability, this membrane permeability is non-selective and may cause damage to healthy cells. The second characteristic is that the receptor is overexpressed on the surface of tumor cells, while it is barely expressed on the surface of normal cells. The receptors overexpressed by tumor cells also have a certain amount of expression in healthy cells, which indicates that healthy cells that can express the same receptor as tumor cells must be affected to a certain extent, so the proportion of receptor expression on the surface of tumor cells and healthy cells is worthy of our attention. The third characteristic is that the number of targeted receptors expressed on the surface of the tumor tissue should reach a certain quantity. The quantity of targeted receptors on the tumor tissue surface, which may be the sum of one or more receptor types, critically determines the number of NCs that can bind. This subsequently has a significant impact on the blood drug concentration. Insufficient receptor numbers might cause the drug concentration to fall below the minimum adequate level, thus compromising the therapeutic efficacy. According to the characteristics of the TME and the obstacles to be overcome to reach the tumor site, we can consider the size, stability, and immunogenicity of ligands, the adaptability of ligands to the receptor topography, the affinity of ligands to the receptor, the chemistry of ligand conjugate sites, the feasibility and cost of ligand construction and other aspects.

**Table 2:** Summary of ligands that enable active targeting.

Targeting molecules	Ligand	Type	Tumor model	Ref.
Targeting cancer-associated fibroblasts	Cys-Arg-Glu-Lys-Ala (CREKA)	Peptide	Breast cancer	[212]
Targeting αvβ3 and αvβ5, integrins overexpressed on angiogenic endothelial cells	iRGD	Peptide	Breast cancer	[213]
Targeting CD44 receptor overexpressed on tumor cells	Hyaluronic acid (HA)	Natural polysaccharide	Breast cancer	[214]
Targeting mitochondria	Alkyltriphenylphosphonium (TPP)	Lipophilic cations	Gastrointestinal cancer	[215]
Targeting folate receptors overexpressed on tumor cells	Folic acid (FA)	Water soluble vitamin	Colon cancer	[216]
Targeting small molecules and proteins to even cells	Aptamers	Single-stranded DNA or RNA molecules	Lung cancer	[217]
Reducing clearance of the phagocytic system and enhancing accumulation at the tumor site	M1 macrophage cell membrane	Biomimetic coating	Breast cancer	[218]
Targeting human epidermal growth factor receptor 2 (HER2) overexpressed on tumor cells	Trastuzumab	Specific monoclonal antibody	Ovarian cancer	[219]
Targeting GRP78 only expressed on tumor cells	Trp-Ile-Phe-Pro-Trp-Ile-Gln-Leu-Lys (WIFPWIQLK)	Peptide	Colon cancer	[220]
Targeting transferrin receptor (TfR) overexpressed in glioma cells and facilitating targeted drug delivery to Glioblastoma (GBM)	HAIYPRH (T7)	Peptide	Glioblastoma	[221]
Targeting epidermal growth factor receptor (EGFR) overexpressed in cancer cells	QRHKPRE	Peptide	Breast cancer	[222]
Targeting p-selectin overexpressed in tumor cells	Taurodeoxycholic acid (TUDCA)	Hydrophilic bile acid	Lung cancer	[223]

Active targeting strategies are usually divided into two types of cellular targets: (1) specific cell surface receptors (such as folic acid, epidermal growth factor receptors, glycoprotein receptors, or overexpressed transferrin) and (2) specific receptors of tumor endothelium (such as vascular endothelial growth factor (VEGF), αvβ3 integrins, vascular cell adhesion molecule-1 (VCAM-1) or matrix metalloproteinases (MMP's)). Active targeting strategies promise greater precision and specific targeting of cancer tissue over passive targeting, which relies solely on EPR effects. NCs become internalized via receptor-mediated endocytosis independent of their surface decorations. Therefore, our improvements should combine active and passive designs. Through active recognition methods, the active design enhances selectivity and specificity, while the passive design, leveraging the EPR effect, devises means to boost drug uptake during dynamic distribution.

However, active targeting also faces many challenges. Regarding ligands, their stability and circulating half-life may be limited, and ligand selection is complex, requiring careful consideration of multiple factors. Additionally, the heterogeneity of tumor cell surface markers increases the selection difficulty. The internal environment significantly impacts it, as tumor vascular barriers in the extracellular matrix can limit the penetration and distribution of NCs in tumor tissues, and protein crown formation can also interfere with targeting. In addition, the diffusion, permeation, and interaction with the microenvironment of nanoparticles in tumor tissues still require further investigation [73]. In clinical translation, actively targeting nanomedicine faces many technical challenges. Its stability is poor and requires strict storage conditions, which is difficult to meet in practical applications, especially in resource-limited areas. The behavior inside the body is complex and challenging to predict, influenced by various factors and differs greatly from *in vitro* experimental results, such as plasma protein interactions, recognition and clearance of the reticuloendothelial system, selective distribution of tumor tissue, and targeting ligands may be affected by the *in vivo* environment and become ineffective. Meanwhile, protein-based active targeted nanomedicines pose immunogenicity risks, which may trigger immune responses, reduce efficacy, and lead to adverse reactions [10]. These all limit the clinical translation of actively targeted nanomedicines.

## 3 Organic nanocarriers

Organic NCs (liposomes, dendrimers, polysaccharides; protein-based NCs) have better biosafety, degradability, and good biocompatibility (biocompatibility refers to the reaction performance of living tissue to inactive substances, that is, whether some materials can be "compatible" with the body or implanted in the body, whether it will cause harm to the human body [81]) compared to inorganic NCs. Among the 15 NCs drugs approved by the FDA and EMA, 12 are organic NCs, accounting for 80 % (Table 1). However, the development of organic NCs is limited by the difficulty of material fabrication and low load capacity [82].

On the one hand, the enhanced biosafety of organic NCs represents a salient advantage. Their biodegradability precludes the accumulation within the body, thereby mitigating the potential for long-term toxicity. Liposomes, for instance, can encapsulate drugs and facilitate their controlled release, which augments the therapeutic effect while minimizing adverse reactions. Doxil® has been efficaciously applied in the treatment of various cancers. In contrast to traditional Doxorubicin preparations, it has manifested a substantially diminished cardiotoxicity and other side effects, concomitantly improving patient tolerance and the overall quality of life [83]. Likewise, DaunoXome® has exhibited unique merits in treating leukemia and other diseases, as it enables drugs to more precisely target cancer cells and reduces damage to normal cells [84]. DepoCyt® can prolong the *in vivo* activity of the drug, enhance its cytotoxicity against tumor cells, and has demonstrated favorable efficacy in treating central nervous system lymphoma and other malignancies [85]. Moreover, Myocet® has played a significant role in the treatment of breast cancer and other cancers by optimizing the pharmacokinetic parameters of the drug and augmenting the therapeutic outcome [86].

Protein-based NCs also show unique advantages. Oncaspar uses protein modification technology to improve the stability and half-life of the drug, which plays an important role in leukemia treatment [87]. Abraxane® and Pazenir ratiopharm combine albumin with paclitaxel to enhance the solubility and targeting of drugs, and they have shown good efficacy in the treatment of various cancers [88]. Apealea® uses polymer micelle technology to optimize paclitaxel's pharmacokinetic and pharmacodynamic properties, providing more treatment options for cancer patients [89].

Nevertheless, the associated challenges cannot be overlooked; the intricate preparation procedures of organic NCs typically necessitate advanced techniques and stringent conditions, which not only escalate production costs but also restrict large-scale manufacturing. Dendrimers involve elaborate steps, including multi-step reactions and precise chemical modifications to regulate molecular size, shape, and surface functional groups. These factors result in a protracted production cycle and demand high technical proficiency in the production environment and from operators. Consequently, the high cost of the product limits its extensive clinical application. Low drug-loading capacity is a problem, requiring more carriers and affecting efficiency. Organic NCs' stability is affected by temperature and pH, leading to drug leakage and reduced efficacy. Additionally, the complexity of the preparation process has been shown to impact the product's quality stability and batch-to-batch consistency in clinical use [90]. The relatively limited drug loading capacity of organic NCs may necessitate frequent or larger administrations in high-dose treatments, inconveniencing patients. In the complex *in vivo* environment, its stability is disturbed by multiple factors that may affect drug release and efficacy. Due to the characteristics of their organic NCs, they also face the problem of cost control and quality optimization in the production process. The stability of the micelles also needs to be strictly controlled under different storage conditions; otherwise, the efficacy of the medicine may be affected [91].

### 3.1 Liposomes

In 1965, Bangham et al. [92] discovered that phospholipids can spontaneously form a vesicle with a double or multilayer structure in water, called liposomes. Liposomes are closed spheroid vesicles composed of an amphiphilic substance with a membrane-like structure. It is surrounded by one or more layers of lipid, with a particle size ranging from tens of nanometers to several microns. Liposome has a lipid bilayer spherical structure with a hydrophilic cavity, the hydrophilic head of the bilayer forms the inner and outer surfaces of the membrane, while the lipophilic/hydrophobic tail end is located in the middle of the membrane. Due to the unique bilayer structure of the liposome, hydrophilic substances are encapsulated in the aqueous lacunae, lipid-soluble/hydrophobic substances are easily embedded in the middle of the bilayer lipid membrane, and amphiphilic molecules are embedded in the interface between the water and the oil phases according to the affinity with the liposome [93]. Due to its unique lipophilicity, liposomes can significantly improve the permeability of drugs to various lipid-based biofilm barriers (such as cell membranes, skin, and blood vessel walls) [94]. Phospholipids have good biodegradability and biocompatibility, so the liposomes composed of phospholipids have good biocompatibility. They can be easily removed from the body, effectively preventing the toxicity caused by the long-term, non-specific accumulation of NCs in normal tissues, so that solves the difficult problem of biodegradability of general NCs [95]. These advantages make liposomes a popular organic NCs. It is worth noting that phospholipids are highly sensitive to the chemical degradation of phospholipids caused by ester group hydrolysis and unsaturated acyl chain oxidation. At the same time, liposomes are a dynamic, thermally unstable system, and the position of phospholipids is constantly switched, resulting in poor physical and chemical stability of liposomes. Improving the stability of liposomes is one of the current research hotspots [96].

**Fig. 5:**
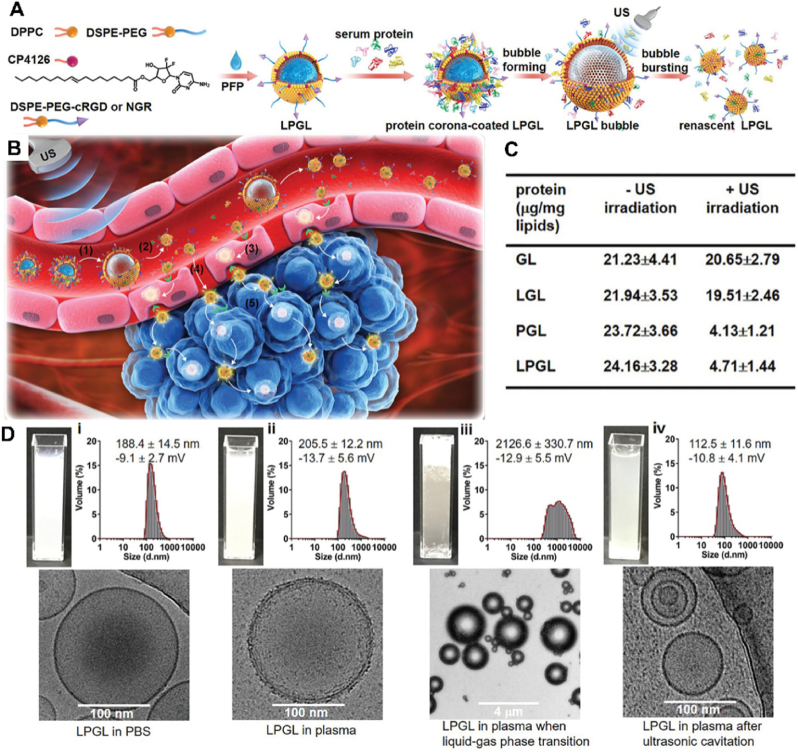
The synthesis process and characterization of liposome LPGL, and schematic diagram of active targeting of LPGL. (A) The process of LPGL involves the composition and fabrication of LPGL, followed by the formation of a protein corona on the liposomal surface, and ultimately results in ultrasonic - cavitation - induced protein - corona decortication. (B) After intravenous injection, LPGL is immediately coated with protein corona in the bloodstream. Once reaching tumor neovascularization, (1) LPGL changes from nano-sized liposomal droplet to microbubble via liquid–gas phase transition by US irradiation; (2) the microbubble breaks into nano-sized liposomes under the prolonged US irradiation, which decorticates the protein corona and discloses the targeting peptides; (3) the exposed targeting peptides recognize and bind to the receptors on the tumor vascular endothelial cells, leading to receptor-mediated transcytosis across the vascular wall and enhanced tumor accumulation; (4) a few of liposomes would also extravasate from blood vessels into tumor periphery via the ultrasonic-cavitation-induced opening of inter-endothelial junctions; (5) the intratumoral liposomes continue to transport deep into the tumor through transcytosis to deliver sufficient gemcitabine and improve therapeutic efficacy. (C) Protein content on the surface of materials before and after ultrasound. (D) Macroscopic images, particle size distribution, and frozen TEM images of materials under different conditions and processing methods.(ⅰ) LPGL in PBS; (ⅱ) LPGL in plasma; (ⅲ) Treat with ultrasound for 20 s at 37 °C; (ⅳ) Treat with ultrasound for 5 min at 37 °C. (A–D) Reproduced with permission [97]. Copyright 2023, John Wiley and Sons.

The phospholipid structure of liposomes can be affected by ultrasonic stimulation [97]. The tiny bubbles in the liquid vibrate, grow, and continuously gather the sound field energy under the action of the ultrasonic field when the ultrasonic energy is high enough. The process of the cavitation bubble collapsing and closing when the energy reaches a certain threshold is called the ultrasonic cavitation effect [98]. After entering the blood circulation, the NCs interact with proteins in the blood and can adsorb proteins to their surface to form a tissue adsorption layer called protein corona (PC) [99]. The coating of PC on the surface of NCs seriously affects the recognition and binding of receptors by ligands of actively targeted therapy strategies, which greatly reduces the efficiency of targeted therapy [100]. Smaller nanoparticles attract relatively few plasma proteins compared to larger nanoparticles [101]. Wang et al. [97] developed a liquid-gas phase variable liposome nanodroplet (Fig. 5A), which can transform nano-liposomes into microbubbles under the ultrasonic cavitation, induce transient or destructive pores on the phospholipid membrane, resulting in changes in the phospholipid structure arrangement of the liposomes and reassembly into smaller nano-liposomes, eliminating or even dissolving the PC (Fig. 5B) so that drugs penetrated and diffuse rapidly. This research consequence demonstrates the feasibility and effectiveness of ultrasound induced liposome recombination strategy, and the liposome without PC is developed successfully. Through the ultrasound cavitation effect of liposomes, we can achieve stable circulation *in vivo* using large-sized NCs. After reaching the target, the cavitation effect turns into tiny bubbles, helping drugs penetrate deep into tissues and cross physiological barriers, solve the problem of non-targeting and the difficulty of deep penetration into tumor tissue.

### 3.2 Polymeric nanoparticles and dendrimers

NCs composed of polymers can achieve precise control of the structure, making them highly flexible. Polymeric nanoparticles (PNPs) potentially exhibit high drug loading, good systemic stability, reduced drug leakage, and controllable drug release. However, these properties are highly contingent on several factors. The nature of the polymer, the solubility and size of the drug molecule, and the fabrication method all have an influence on PNP drug loading capacity. For example, hydrophobic polymers suit hydrophobic drugs, while hydrophilic polymers benefit water-soluble drugs. PNPs' systemic stability is affected by surface characteristics, like hydrophilic coatings (e.g., polyethylene glycol), which enhance resistance to opsonization and phagocytosis. Their release performance is governed by polymer composition (e.g., biodegradable linkages), environmental stimuli (pH, temperature, enzymatic activity), and nanoparticle morphology. Thus, PNPs' performance in these properties varies significantly with specific circumstances and design parameters [102]. Biocompatible polymeric carriers used in the medical field include polyester, polyethylene glycol, chitosan, polyurethane, polyamide, polyanhydride, polylactic acid hydroxyacetic acid copolymer, etc. [103,104].

Dendrimers are highly branched, three-dimensional, monodispersed synthetic macromolecules with a dendritic structure. Dendrimers are synthesized by a stepwise polymerization reaction that consumes repeat units in the tangled products. The structure of a dendrimer consists of the core, containing multiple repeat units in a branched polymeric solid and capped by a shell covered with surface functional groups. The nanoparticle has an extremely low polydispersity coefficient because its molecular weight and particle size can be precisely controlled. At the same time, a large number of dendritic terminal groups distributed on the surface of its three-dimensional structure provide sites for attachment of targeting ligands. The attachment of ligands often requires some particular surface modification, although its internal core structure contains porous cavities that can be manipulated to form a unique microenvironment for transporting drugs [105]. These properties, therefore, permit their use as drug carriers, which transport drugs and nucleic acids to localized targets. By linking appropriate targeting ligands to their surfaces, they become efficient and selective transporters of drugs to cellular targets without affecting normal cells.

**Fig. 6:**
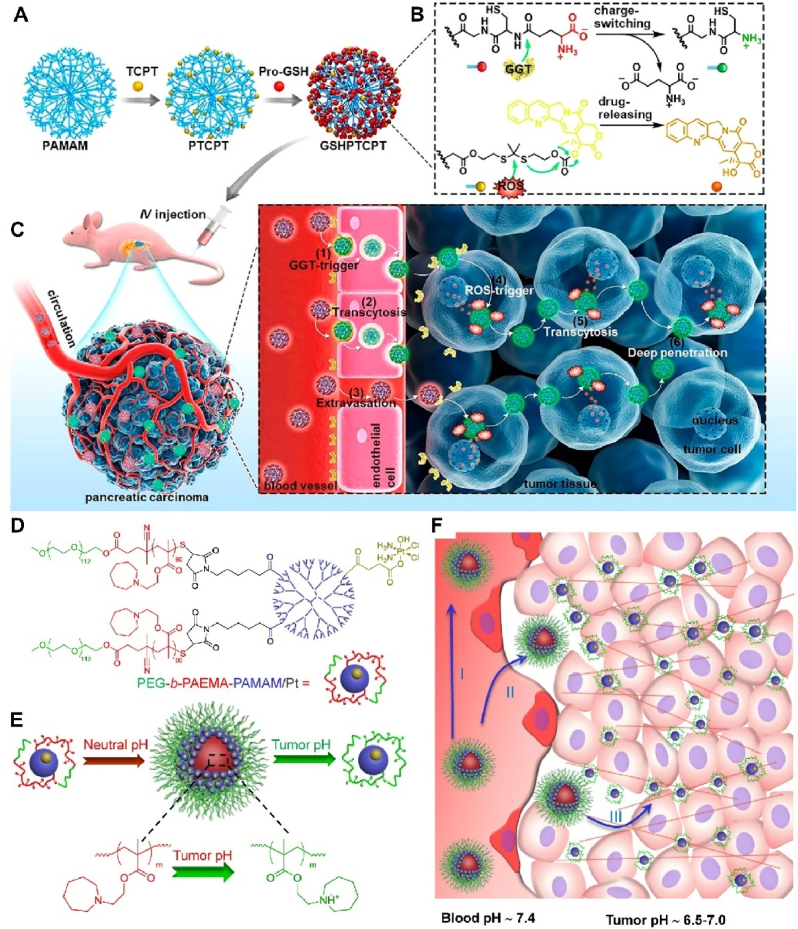
Targeted therapy of cancer with dendrimers. (A) Schematic diagram of the synthesis of dendrimer camptothecin conjugate (GSHPTCPT). CPT is covalently linked to polyamide amide (PAMAM) dendrimers via reactive oxygen species (ROS) sensitive linkers and then surface modified with glutathione to synthesize dendrimers drug conjugates. (B) The reaction mechanism diagram of GGT catalyzed glutamyl charge reversal, and ROS triggered GSHPTCPT cleavage to release CPT. (C) Schematic diagram of the mechanism of GSHPTCPT *in vivo*. Once the conjugate is delivered to the periphery of PDA tumors, overexpressed GGT on vascular endothelial cells or tumor cells triggers γ-glutathione glutamyl transfer reaction to produce primary amines. The positively charged conjugate rapidly internalizes through endocytosis mediated by small pits and then enhances its deep penetration into the tumor parenchyma through vesicle-mediated trans endocytosis, releasing active CPT throughout the entire tumor after intracellular ROS lysis. (A–C) Reproduced with permission [106]. Copyright 2020, American Chemical Society. (D) Structure of PEG-b-PAEMA-PAMAM/Pt. (E) PEG-bPAEMA-PAMAM/Pt self-assembling into SCNs/Pt at neutral pH, and the degradation of SCNs/Pt into small particles at tumor acid pH.(F) Schematic diagram of the *in vivo* mechanism of action of PEG-b-PAEMA-PAMAM/Pt. (D–F) Reproduced with permission [107]. Copyright 2016, American Chemical Society.

The dense fibrous stroma in pancreatic ductal adenocarcinoma (PDA) hinders the entry and diffusion of drugs into tumor tissue, leading to poor therapeutic efficacy. Therefore, Wang et al. [106] developed a dendritic polymer camptothecin (CPT) conjugate, which can actively target and penetrate deep into PDA by γ-glutamyltranspeptidase (GGT) -triggered endocytosis and transendocytosis. CPT is covalently linked to polyamide amide (PAMAM) dendrimers via reactive oxygen species (ROS) sensitive linkers. Then, the surface is modified with glutathione to synthesize dendrimer drug conjugates (Fig. 6A). Once the conjugate is delivered to the periphery of PDA, overexpression of glutamyltranspeptidase (GGT) on vascular endothelial cells or tumor cells trigger γ-glutamyl transfer of glutathione which produces primary amines. The positively charged conjugate rapidly internalizes through endocytosis mediated by small pits and then enhances its deep penetration into the tumor parenchyma through vesicle-mediated trans endocytosis. After intracellular ROS lysis, it releases active CPT throughout the tumor (Fig. 6B and C). Li et al. [107] reported a pH-responsive nanoparticle superstructure formed by the directional assembly of an ionizable tertiary amine group containing an amphiphilic polymer and a platinum-containing prodrug; both were conjugated to a polyamidoamine (PAMAM) and PAEMA dendrimer (Fig. 6D). To increase tumor penetration and kill efficacy *in vivo*, an ultra-sensitive trigger that initiates size reduction in an acidic TME was necessary. At neutral pH (such as blood circulation), these superstructures start at an initial size of 80 nm, but once they localize in the slightly acidic TME (pH 6.5 to 7.0), they go through a dramatic size transformation within a range of less than 0.1–0.2 pH units. By affecting this change, the 80 nm nanoparticle quickly hydrolyzes into 10 nm-diameter dendritic building blocks. Rapid size switching allows for deeper tumor penetration and accumulation in tumor tissue through passive targeting. This is aided by faster nanoparticle diffusion (Fig. 6E and F). This study shows that pH-triggered size switching is a workable method to increase the permeability and bioavailability of medications and improve therapeutic effectiveness.

### 3.3 Polymeric nano micelle

Polymer nano micelles (PMs) are typically a type of nanoparticle with a unique core-shell structure formed by self-assembling amphiphilic polymers in specific solvents. They are composed of a hydrophobic core and a hydrophilic shell, with a particle size of generally 10–200 nm, and are prone to loading hydrophobic drugs [108]. The hydrophilic shell of PMs can effectively increase the circulation time of the carrier in the body, thereby extending the half-life of the drug [109]. Moreover, some micelles based on biodegradable and well-biocompatible polymers are not easily phagocytic by the reticuloendothelial system (RES) and can effectively prevent the adsorption of proteins and cells. Such as polylactic acid - glycolic acid copolymer (PLGA), polycaprolactone (PCL), etc., the metabolic process of these polymer micelles in the body is more compatible with the physiological process of the body, and it is not easy to trigger the immune response of the body, thus reducing the probability of being recognized and swallowed by RES [110]. Therefore, it can circulate in the blood for a long time and remain stable. The speed and amount of drug release can be controlled by adjusting the type and structure of the polymer [111]. Polymeric materials used for preparing nano micelles include poly (alkylethylene glycol), poly (lactic acid), propylene glycol copolymer (PLAG), and poly (alkyl cyanoacrylate).

**Fig. 7:**
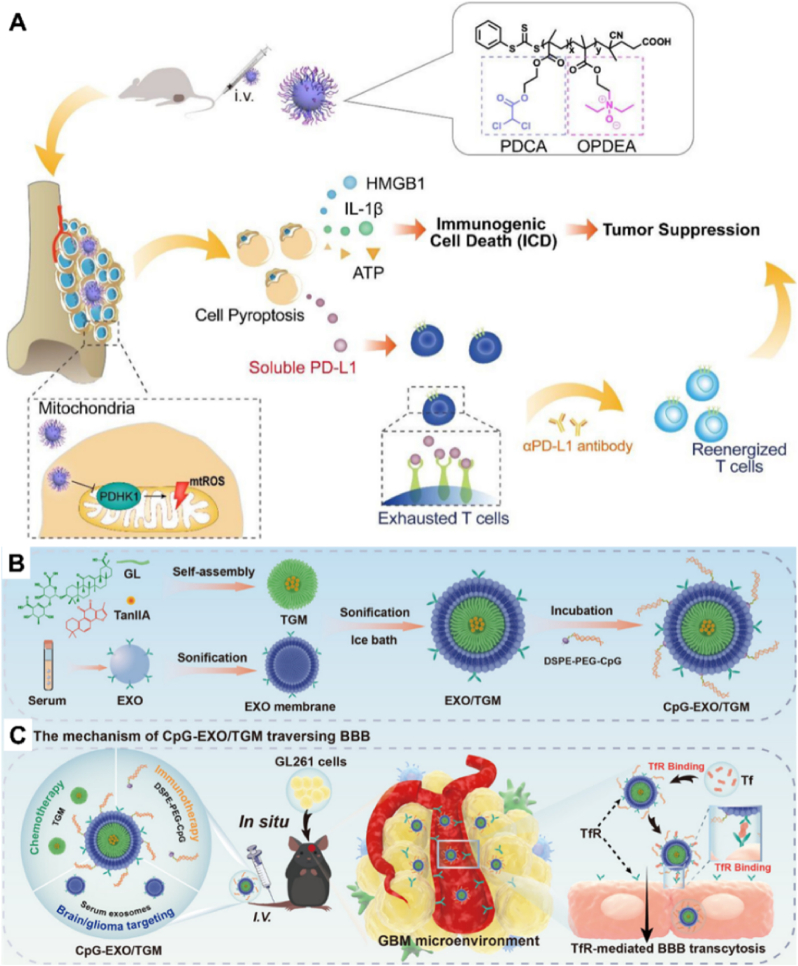
Application of polymeric nano micelles in targeted therapy. (A) Structure of OPDEA-PDCA. OPDEA-PDCA can target mitochondria and induce mitochondrial oxidative stress by inhibiting PDHK1, leading to immunogenic cell apoptosis in osteosarcoma cell lines and thus inhibiting tumors. Meanwhile, OPDEA-PDCA can induce the secretion of soluble programmed cell death ligand 1 (PD-L1) molecules, consuming T cells. The combination therapy of OPDEA-PDCA and anti-PD-L1 monoclonal antibody significantly inhibits the proliferation of osteosarcoma and prolongs T cell activation. Reproduced with permission [44]. Copyright 2022, American Chemical Society. (B) The synthesis scheme of CpG EXO/TGM. TanIIA GL nano micelles (TGM) are self-assembled through hydrogen bonding and hydrophobic interactions between TanIIA and amphiphilic glycyrrhetinic acid (GL). Endogenous serum exosomes encapsulate pure drug nano micelles, anchor CpG oligonucleotides onto exosome membranes, and construct immune exosomes loaded with self-assembled traditional Chinese medicine nano micelles (CpG EXO/TGM). (C) Mechanism of CpG EXO/TGM crossing BBB. The TfR on the surface of CpG EXO/TGM can bind to the overexpressed transferrin (Tf) on the surface of BBB and GBM, enabling the nanocarrier to target BBB and GBM cells and penetrate BBB through the intracellular pathway mediated by TfR. Reproduced with permission [112]. Copyright 2023, American Chemical Society.

The technology of self-assembly of nano micelles has received much attention in the field of drug delivery. According to reports, cell apoptosis can improve the immunosuppressive tumor microenvironment and may be a strategy for enhancing the treatment of osteosarcoma. The extent to which mitochondrial regulation can induce tumor cell apoptosis to enhance the characteristics of immunotherapy has not been determined. Jin et al. [44] synthesized a mitochondrial-targeted PMs (OPDEA-PDCA) using poly [2- (N-oxide-N, N-diethylamino) ethyl methacrylate] (OPDEA) targeting mitochondria and conjugated dichloroacetate (DCA) for inhibiting pyruvate dehydrogenase kinase 1 (PDHK1). According to this study, OPDEA-PDCA targeted mitochondria and induced mitochondria and induce mitochondrial oxidative stress by inhibiting PDHK1, leading to immunogenic pyroptosis in osteosarcoma cell lines. In addition, researchers have found that OPDEA-PDCA can induce the secretion of soluble programmed cell death ligand 1 (PD-L1) molecules. Therefore, the combination therapy of OPDEA-PDCA and anti-PD-L1 monoclonal antibody can significantly inhibit the proliferation of osteosarcoma and prolong T cell activation (Fig. 7A). This study provides a strategy to initiate cell pyroptosis by targeting mitochondria, which may combine with immunotherapy to enhance anti-tumor efficacy. Cui et al. [112] were inspired by small molecule self-assembly technology and used tanshinone IIA (TanIIA) extracted from traditional Chinese medicine (TCM) to induce tumor cell apoptosis and inhibit neovascularization, as well as amphiphilic glycyrrhetinic acid (GL) for hydrogen bonding and hydrophobic interaction self-assembly, to prepare TanIIA-GL nano micelles (TGM). Subsequently, endogenous serum exosomes were selected to encapsulate the pure drug nano micelles and the agonist CpG oligonucleotide of toll-like receptor 9 was anchored onto the exosome membrane to construct an immune exosome (CpG EXO/TGM) loaded with self-assembled traditional Chinese medicine nano micelles (Fig. 7B). The research results indicate that the TfR on the surface of CpG EXO/TGM can bind to the overexpressed transferrin (Tf) on the surfaces of BBB and GBM, enabling the NCs to target BBB and GBM cells and rely on the TfR mediated intracellular pathway to penetrate BBB (Fig. 7C).

## 4 Inorganic nanocarriers

Inorganic NCs have been synthesized from various materials such as mesoporous silica NCs, gold NCs, clay minerals, and quantum dots, achieving variable success. Compared to organic NCs, their synthesis methods are relatively simple, and they are prepared in different shapes and sizes to deliver chemotherapy drugs to tumors under specific conditions. Some inorganic NCs can also provide electrical, magnetic, and optical properties. Although inorganic nanoparticles are easy to regulate, their biocompatibility is poor and difficult to degrade. Generally speaking, their dispersibility is poor. These fatal drawbacks seriously hinder the development of clinical applications of inorganic NCs and are worth overcoming by researchers. Currently, only two inorganic NCs drugs have been approved by the FDA or EMA, NanoTherm® And Hensify.

NanoTherm® has demonstrated certain potential in cancer treatment. It exploits the magnetism of the nanoparticles to achieve targeted drug delivery and therapy guided by magnetic resonance imaging [113]. Theoretically, this enables drugs to be more precisely localized within tumors, enhancing the treatment effect while minimizing harm to healthy tissues. Nevertheless, the practical application of "nanothermal therapy agents" has been obstructed by several key issues. The biocompatibility of inorganic NCs is a significant concern. The accumulation of non-degradable nanoparticles in the body raises the possibility of immune responses and long-term toxicity, which cannot be overlooked. In clinical settings, this may pose unexpected complications and risks to patients. Another notable drawback is its poor dispersibility. During the formulation and *in vivo*, nanoparticles aggregate, resulting in unstable drug release and diminished efficacy. The magnetic properties, intended to confer advantages, actually exacerbate the aggregation tendency, especially in the presence of external magnetic fields. In clinical applications, any variations in the properties of nanoparticles can directly impact treatment outcomes, presenting numerous challenges for translating this technology from the laboratory to clinical practice.

Hensify (NBT XR3), approved by the EMA in 2019, is not as extensively studied as some more established NCs but represents a new entrant in this field [91]. Currently, information regarding its full potential and limitations remains relatively scarce. Preliminary studies suggest that it might offer certain benefits in cancer treatment, perhaps through a novel mechanism of action or more efficient drug delivery strategies. However, like many emerging treatment modalities, it also confronts substantial obstacles in its journey toward clinical application. A significant hurdle is the lack of comprehensive data on its long-term safety and efficacy. Without a clear understanding of its interaction with the human body over time and potential side effects, it is difficult to ascertain its actual value in patient treatment. In conclusion, inorganic NCs have exhibited certain promise in the cancer treatment domain, but their existing limitations in biocompatibility, dispersibility, and production scalability pose significant challenges for their clinical translation. Researchers must address these issues through meticulous experimentation and innovation to ensure that these potential treatment methods can be safely and effectively implemented in patient care while adhering to the principles of medical ethics and patient well-being [114].

### 4.1 Quantum dots

**Fig. 8:**
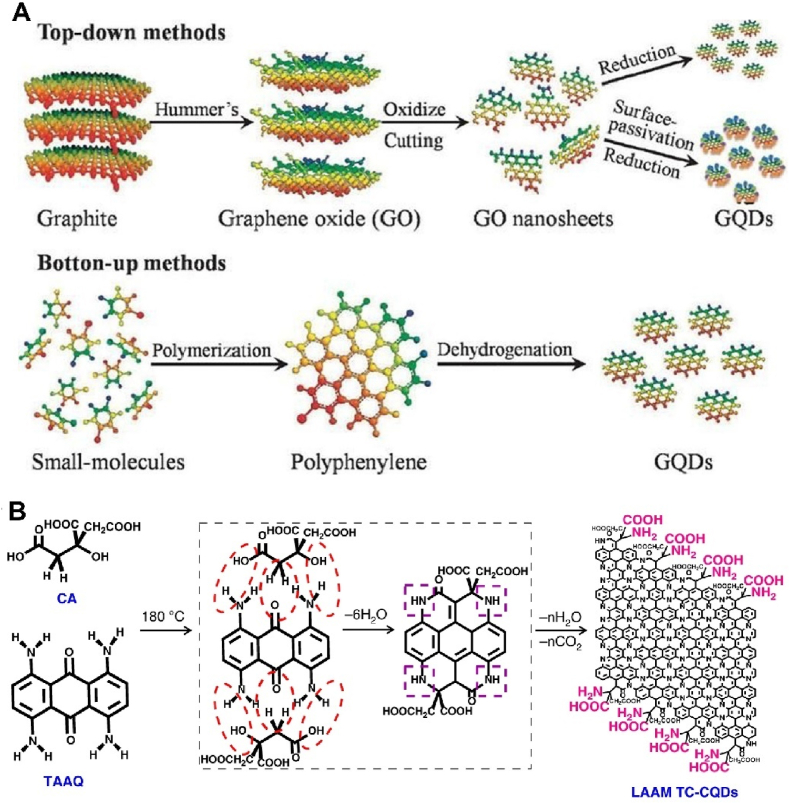
Synthesis method of quantum dots and application of drug delivery. (A) The two main ways to prepare GQDs are the “top-down” cracking method using different carbon sources and the “bottom-up” cracking method using small molecules or polymers. Reproduced with permission [123]. Copyright 2012, Royal Society of Chemistry. (B) Diagram of the preparation method for LAAM TC-CQDs. The structure of amino acids was simulated using carboxyl and amino functionalized CQDs, achieving multifunctional integration of near-infrared imaging, photoacoustic therapy, and chemotherapy drug delivery. Reproduced with permission [124]. Copyright 2020, Springer Nature.

Quantum dots (QDs) are semiconductor particles that appear at the nanometer level in all three dimensions, with sizes as small as 1–20 nm [115]. They are also known as "artificial atoms" or "artificial atoms" [116]. The shape of QDs can be elliptical, triangular, square. The dot of QDs mainly refers to its small spatial size. Quantum primarily refers to the quantum confinement effect that begins to form as the size decreases, allowing for quantum control of the observed properties of these substances [117]. The commonly used quantum dot materials in the field of targeted drug delivery currently include carbon quantum dots (CQDs) [117], graphene quantum dots (GQDs) [118], phosphorus quantum dots (PQDs) [119], Mxene quantum dots (MQDs) [120] etc. They possess the dual excellent properties of quantum dots and the components of the material itself, with excellent optical properties, high specific surface area, large π - π-conjugated systems, and modifiable edge groups [121]. Therefore, it can achieve good photothermal conversion ability and ultra-high drug loading rate. In addition to the field of drug delivery, they are also popular materials for constructing biomedical fluorescence sensors, which can achieve tumor visualization and treatment and exhibit good stability, water solubility, and chemical inertness [122]. GQDs with adjustable size can generally be prepared using top-down and bottom-up methods (Fig. 8A) [123]. The top-down approach refers to separating and cutting graphene flakes into GQDs, which includes chemical ablation, electrochemical oxidation, and oxygen plasma treatment. The bottom-up approach includes synthesizing graphene fragments containing a certain number of conjugated carbon atoms, such as the cage opening method of fullerenes or the solution chemistry method of forming GQDs from molecular precursors. Usually, these GQDs have surfaces rich in carboxylic acid functionality and can be used to bind surface passivating agents. It is an excellent drug carrier. Li et al. [124] utilized α- Carboxyl and amino functionalized CQDs mimic the structure of amino acids, successfully preparing a multifunctional nanoplatform for near-infrared imaging, photoacoustic therapy, and chemotherapy drug delivery (Fig. 8B). It can specifically bind to the large neutral amino acid transporter 1 expressed in tumors, thereby targeting the tumor site. This research achievement demonstrates the powerful advantages of QDs in tumor specific imaging and drug delivery. Their structure and composition are easy to control, and they can load aromatic drugs through π - π stacking interactions. They have ideal biocompatibility and are a very promising class of multifunctional biopharmaceutical materials.

### 4.2 Nano-clay minerals

**Fig. 9:**
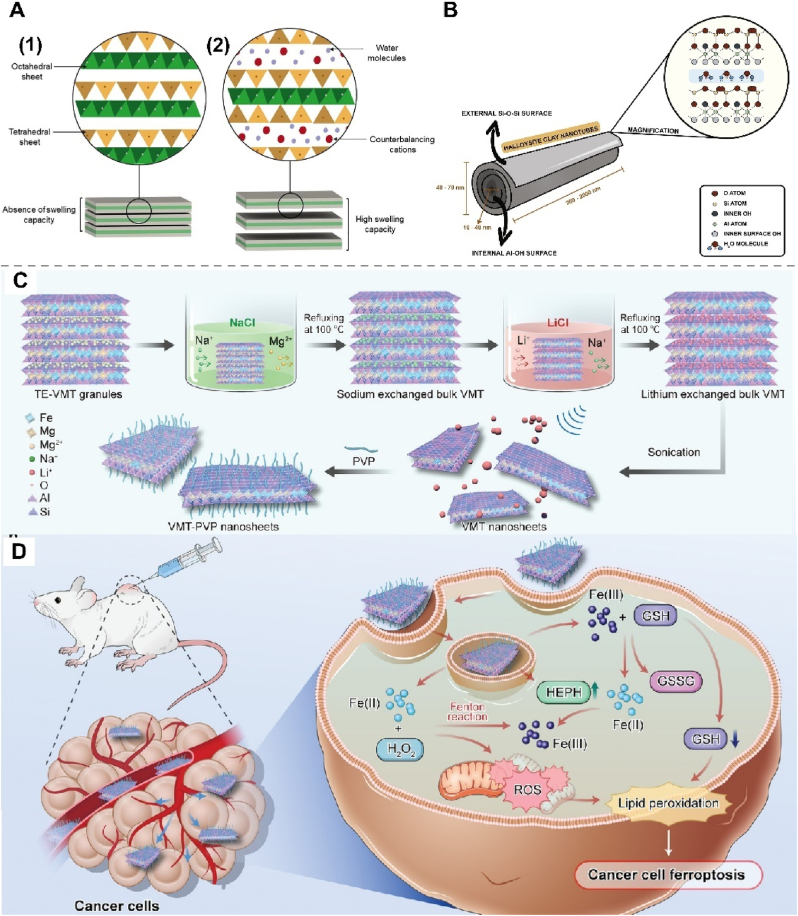
Nano-clay minerals for targeted therapy of tumors. (A)Two structures of layered nano clay. (1) 1:1 type of nano clay; (2) 2:1 type of nano clay. Reproduced with permission [128]. Copyright 2021, Elsevier. (B) Schematic diagram of the structure of the halloysite. Reproduced with permission [129]. Copyright 2021, Elsevier. (C) Fabrication process of VMT-PVP nanosheets. (D)When endogenous H_2_O_2_ is converted to ·OH through the oxidation-reduction reaction of VMT-PVP nanosheets and overexpressed GSH, additional intracellular ROS is generated, and lipid peroxidation (LPO) levels are upregulated, inducing iron deficiency anemia and leading to enhanced CDT effect. (C–D) Reproduced with permission [138]. Copyright 2022, John Wiley and Sons.

Natural aluminosilicate clay minerals have attracted much attention in cancer diagnosis and treatment research due to their low price, good biocompatibility, strong cation exchangeability, adjustable morphology, high specific surface area, and strong adsorption capacity [125]. Since the beginning of the 20th century, clay minerals have been included in the Western Pharmacopoeia as pharmaceutical products. Among them, two-dimensional layered nanoclay such as montmorillonite, kaolinite, illite, and tubular nanoclay (halloysite) are receiving increasing attention in drug delivery [126]. They can be electrically neutral or carry negative charges. Clay minerals can be divided into 1:1 and 2:1 types according to crystal structure [127]. 1:1 type minerals, such as kaolin, exhibit electrical neutrality. Due to the strong bonds between the aluminum octahedral and silicon tetrahedral sheets, the structure is relatively tight, resulting in the absence of any equilibrium cations in the interlayer space; Therefore, these types of clay minerals are not hydrated and have almost no swelling properties (Fig. 9A). The charge of minerals with a crystal structure of type 2:1, such as montmorillonite, is variable. This structure forms weak bonds between the two layers of silicon-oxygen tetrahedral sheets. If the structure has a negative charge, there may be equilibrium cations in the interlayer space; If the structure is neutral, it may not contain any equilibrium cations. Therefore, negatively charged structures can be hydrated and exhibit expansion properties [128], Halloysite belongs to a 1:1 type mineral with a unique tubular structure. Although halloysite is similar to kaolinite, it contains water molecules in the interlayer space. The transverse mismatch between octahedral and tetrahedral sheets facilitates the rolling of halloysite, resulting in porous spiral multi-walled tubes with intermediate cavities. Due to different mineral sources and viscosity characteristics, the length of HNTs is generally 200nm-2 μ m. The inner diameter of the tube is 10 nm–40nm, and the outer diameter is 40 nm–70nm (Fig. 9B) [129]. Through some simple treatment methods (calcination [130], acid/alkali leaching [131], intercalation [132], exfoliation [133], surface modification, [134]) [127] the physical and chemical properties of layered nano clay can be easily changed to improve the ability of drug loading and targeted delivery. The physical and chemical properties of nanotubes can be enhanced by adjusting the inner and outer tube diameter, edge surface, pore structure, and inner layer structure [135].

Vermiculite (VMT) is a 2:1 type clay mineral that expands in high-temperature environments. When suddenly heated to 200–300 °C, VMT produces peeling along the c-axis of its crystal, and its volume can rapidly expand several to tens of times. VMT is a commonly used traditional Chinese medicine herb with excellent biocompatibility [136]. Due to the high content of Fe (III) in the structure of VMT, it has great potential in cancer treatment. VMT-based nanoplatforms can utilize Fe (III) to consume GSH to mediate TME regulation and produce Fe (II) to induce the Fenton reaction that generates OH [137]. Then, it disrupts the antioxidant activity of cancer cells. At the same time, a large amount of GSH is consumed, leading to an increase in intracellular reactive oxygen species content and lipid peroxidation, inducing cell apoptosis, also known as ferroptosis. Ma et al. [138] utilized the aforementioned properties of vermiculite and prepared ultra-thin 2D vermiculite (VMT) nanosheets from thermally expanded vermiculite (TE-VMT) crystals using countercurrent ion exchange and a top-down approach (Fig. 9C). Polyvinylpyrrolidone (PVP) modified VMT-PVP nanosheets have good stability. It can consume GSH and provide Fe (III)/Fe (II) ions, disrupting the body's antioxidant defense and achieving ferroptosis-based chemodynamic therapy (CDT). VMT-PVP has good biocompatibility and can target TME overexpressing GSH. When endogenous H_2_O_2_ is converted to · OH through the oxidation-reduction reaction of VMT-PVP nanosheets and overexpressed GSH, additional intracellular ROS is generated, and lipid peroxidation (LPO) levels are upregulated, inducing iron deficiency anemia and leading to enhanced CDT effect (Fig. 9D). This work utilizes the synergistic effect of iron apoptosis induced by the consumption of GSH Fenton reaction by a large amount of Fe (III) in vermiculite to achieve significant anti-tumor effects *in vivo*, while having almost no effect on normal tissues, indicating the application of nano mineral materials in the field of tumor treatment. The in-depth study of the characteristics of natural nano-mineral materials is of great significance in biomedicine and material engineering. For example, vermiculite can induce tumor ferroptosis, and montmorillonite can adhere to the intestine and resist acid, which is beneficial for gastrointestinal administration; the unique tubular structure of halloysite can better protect drugs [128].

### 4.3 Mesoporous silica nanoparticles

**Fig. 10:**
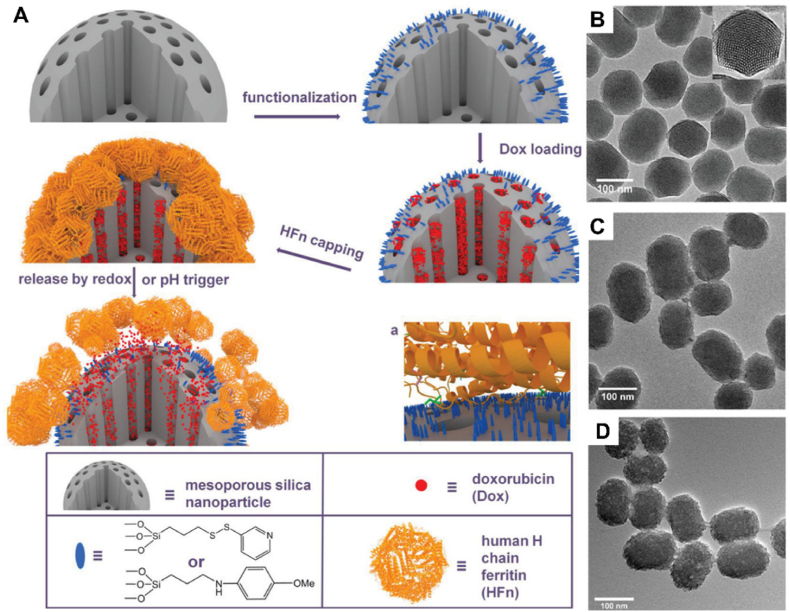
Schematic illustration of drug delivery systems with MSNs. (A) HFn bonds to the functionalized MSN via disulfide or hydrophobic interactions, and HFn blocks the pores of MSN. The link between MSNs and HFn is sensitive to oxidation-reduction or pH changes. (B) TEM image of synthesized MSNs. The enlarged insertion image clearly displays the pores of MSN. (C) TEM images of oxidation-reduction sensitive nanovalve encapsulated HFn microspheres. (D) TEM images of pH sensitive nanovalve encapsulated HFn microspheres. (A–D) Reproduced with permission [141]. Copyright 2020, John Wiley and Sons.

Mesoporous silica nanoparticles (MSNs) have become one of the most popular NCs for drug-targeted delivery due to the following advantages [139]: (1) Due to the extremely high specific surface area and porous structure, MSNs have strong drug-loading ability. (2) The pore size of MSNs is adjustable, and its high specific surface area makes it easy to modify various functional groups on the surface so that we can graft targeting ligands efficiently to achieve active targeting. (3) MSNs have good physiological stability and biocompatibility. However, relying solely on MSNs to load therapeutic molecules and grafting targeted ligands for active targeting strategies cannot meet existing clinical problems. For example, before delivering therapeutic molecules to the lesion site, the therapeutic molecules are prone to early release from the pores of MSNs [140,141], the surface modification of targeted ligands is off target [142], a single treatment method has insufficient anti-tumor effect and poor biodegradability [143]. These issues need to be considered in the design of silicon-based drug delivery systems. Glutathione (GSH) is a protein that contains γ- Amide bonds and thiol tripeptides in almost every cell. GSH has two forms: reduced form (G-SH) and oxidized form (G-S-S-G), with reduced glutathione accounting for the majority under physiological conditions. The concentration of reduced GSH in the TME is 4–7 times higher than that of normal cells, and the concentration of GSH in human plasma is very low, providing a suitable environment for reduction-sensitive release. Upon exposure to reduced glutathione (GSH), disulfide bonds (-S-S-) exchange with the thiol groups present in GSH. This reaction has been widely exploited in drug delivery to engineer redox-responsive systems, enabling precise control over drug release kinetics [144]. Human H-chain ferritin (HFn) can specifically bind to transferrin receptor 1 (TfR1, a receptor overexpressed in various tumors) [145]. To solve the problems of early release and non-specific drug loading in MSNs, Cai et al. [141] used HFn as a cap to seal the pores of MSNs and prepared two kinds of cleavable linkers to connect HFn and MSNs (Fig. 10A and B). The first use of disulfide bonds as linkers, due to the redox sensitivity of disulfide bonds, when the NCs encounter high concentrations of glutathione (GSH) in the TME, HFn can be dissociated through the reduction of disulfide bonds, followed by the release of drugs, making the NCs system capable of redox response (Fig. 10C). The other is the use of aromatic amine stalk as a connector when reaching the acidic TME, the aromatic amine stalk will be protonated, allowing the loaded drug to be released (Fig. 10D). These two experiments solved the obstacles of early drug release and non-specific drug delivery of MSNs, providing ideas and references for drug delivery using nano mesoporous materials.

### 4.4 Metal nanocarriers

Precious metal materials (Au, Ag, Pt, Pd) have excellent photothermal conversion efficiency and stability [146]. Photothermal therapy (PTT) harnesses the photothermal effect of photothermal reagents (PTA) for transforming NIR light with good tissue penetrability into heat. Thereby, it increases the local temperature of the tumor and detriments the normal function of tumor cells, ultimately leading to apoptotic or necrotic cell death [147]. The inorganic PTA with the most research on precious metal materials. Precious metal materials can absorb photons to activate ground-state electrons to excited states and then release heat energy through non-radiative decay [148]. Among these materials, gold nanoparticles are particularly prominent, with excellent photothermal conversion efficiency, easy surface modification, good biocompatibility, and adjustable absorption wavelength, which can be easily adjusted to the near-infrared region [149]. Gold nanomaterials have free electrons on their surface [150].When the wavelength of incident light resonates with the vibration frequency of free electrons, surface plasmon resonance (SPR) is generated [151]. The SPR wavelength of gold nanomaterials can be adjusted between the near-infrared region (700 nm–1300 nm) by changing the aspect ratio of the particles [152]. A near-infrared laser can transmit blood and tissue to the maximum extent, and perform photothermal therapy on deep tumor tissue [67]. Therefore, gold nanomaterials are often used for photothermal therapy of tumors and have become one of the hot topics in photothermal anti-tumor materials. However, due to the limited depth and range of light penetration into tissues, tumors outside light irradiation cannot be completely ablated [153]. The low delivery efficiency of PTA and the excessively high temperature in the tumor area, which can damage normal cells, limit the treatment efficiency of PTT [154]. Moreover, PTA based on precious metals faces with the problem of expensive and difficult to degrade [155], and clinical applications must consider these issues. Using nanoparticles as drug carriers and combining PTT with targeted therapy can improve the aforementioned issues. In addition, there is also Multisite targeting [156,157], Immune-targeting combination therapy [158,159], Radio-targeted combinationtherapy [56,160], co-delivery strategies of multiple therapeutic molecules [161] and photodynamic-targeted combination therapy [162] to improve the efficiency of tumor treatment.

Peritoneal metastasis (PM) is considered the advanced stage of metastatic colon cancer, where malignant tumors develop metastatic lesions, and the lesions migrate to the peritoneal area. Even with the latest and best chemotherapy regimens, median survival is still low [163]. The current PM treatment combines cell reduction surgery (including removal of all visible tumors) and hyperthermic intraperitoneal chemotherapy (HIPEC), which utilizes hyperthermia to promote the diffusion and cytotoxicity of chemotherapy drugs [164]. Since HIPEC is carried out through closed circulation of hot liquid containing chemotherapy, it will lead to uncontrolled heating and drug distribution in the whole abdominal cavity, which has important off-site toxicity and a high incidence rate. Based on the above clinical obstacles, Florence Gazeau et al. [165] utilized a safer and more precise strategy of photothermal targeting, using near-infrared (NIR) light-activated gold nanoparticles (AuNPs) coupled with chemotherapy drug 5-fluorouracil (5-FU), to achieve spatial and temporal control of targeted intraperitoneal tumor nodules and mild chemotherapy hyperthermia through intraperitoneal administration. Compared to a single PTT, the near-infrared photothermal therapy involving 5-FU provides additional anti-tumor effects, which solves the problem of the treatment efficiency of PTT is insufficient due to the limited light penetration ability. Intraperitoneal injection of AuNP-5-FU preferentially accumulates in tumor nodules and peritoneal macrophages, improving the precise treatment level of PTT, prolonging tumor necrosis, and activating pro-inflammatory immune responses while not damaging healthy tissues. This combination therapy can potentially overcome the current non-targeted toxicity of HIPEC in clinical practice. Utilizing the unique properties inherent in metal nanomaterials makes it easy to develop combination therapy strategies to help us efficiently combat tumors.

In addition to the high specific surface area, excellent stability, and good biosafety, gold nanoparticles have special size-dependent optoelectronic properties and good catalytic ability. Gold nanorods are another relatively common gold nanomaterial with the same stability and biosafety characteristics as gold nanoparticles. The difference is that gold nanorods have a richer set of unique physical properties, with two plasmon resonance peaks in the transverse and longitudinal directions and peak resonances that can be adjusted by adjusting the aspect ratio of the entire gold nanorod over an extensive range. They respond from the visible to the near-infrared region when small changes are made in the local environment and convert such changes into detectable optical or electrical signals. Thus, gold nanorods demonstrate a wide range of applications in nanobiology, such as molecular probes, biosensing, and cellular imaging. In addition, gold nanorods absorb near-infrared light and convert it rapidly to heat through non-radiative processes with high photothermal conversion efficiency. This property has made them useful for photothermal therapy. Due to its deeper tissue penetration and higher imaging contrast, research focuses on creating diagnostic compounds for the NIR-II region. Gold nanocages demonstrate excellent imaging capabilities and the ability to efficiently absorb photons and transform light energy into localized hot spots [166]. These methods and their implementation suggest new possibilities that confer extra selectivity by irradiation with directed light beams as the energy source for dual PTT and PDT. In addition, due to their tunable porous hollow structure, gold nanocages have also been used as drug carriers.

**Fig. 11:**
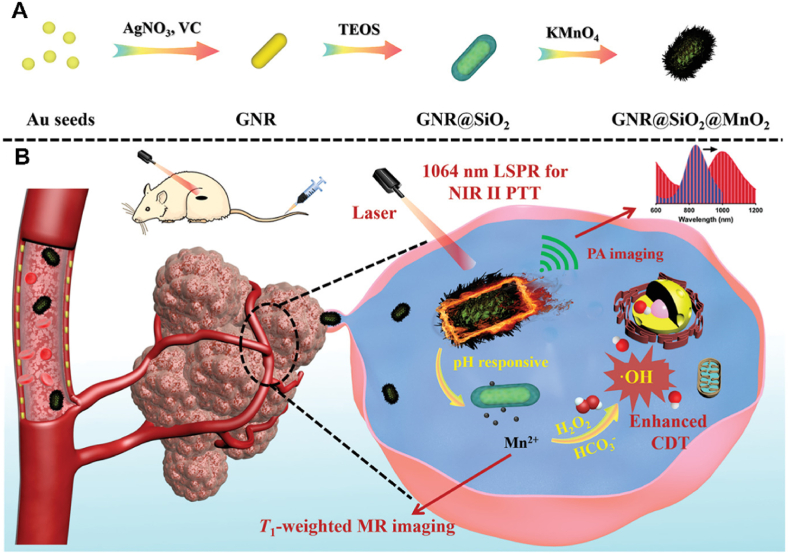
The synthesis method and antitumor mechanism of GNR@SiO_2_@MnO_2_. (A) Schematic diagram of the preparation process of GNR@SiO_2_@MnO_2_. (B) The anti-tumor mechanism of GNR@SiO_2_@MnO_2_. (A–B) Reproduced with permission [167]. Copyright 2021, John Wiley and Sons.

He et al. [167] developed a MnO_2_-coated, Goldnanorods plasma modulation scheme to encode photoacoustic (PA) and magnetic resonance (MR) signals for NIR-II pumped photo thermochemical kinetic therapy (Fig. 11A). The double signal encoding allows the collection of dual PA and MR images that are responsive to the TME (Fig. 11B). By consisting of the total sandwich structure GNR@SiO_2_@MnO_2_, the localized surface plasmon resonance peak is adjusted to resonate over a range between the NIR-I to the NIR-II region. The MnO_2_ layer was degraded, and the GNR@SiO_2_@MnO_2_ revealed TME reactive photoacoustic and magnetic resonance imaging. Additionally, Mn^2+^ released from GSM after degradation of the MnO_2_ layer catalyzes the creation of the lethal hydroxyl radical (•OH) from H_2_O_2_ under acidic conditions of the TME. These combination processes constitute the essential elements of chemodynamic treatment (CDT) of tumors.

## 5 Hybrid nanocarriers

Organic NCs have good biocompatibility and safety, but their stability, controllability, and modification are inferior to those of inorganic NCs. They easily degrade or change their structure in complex physiological environments, making regulating their properties difficult to regulate. Although inorganic NCs have unique acoustic and optical properties, their direct application is limited due to poor biocompatibility. They may trigger immune responses and toxicity issues. Hybrid NCs integrate the advantages of both; however, their development faces many challenges, such as complex synthesis, high cost, and poor interface compatibility, which affect their large-scale production and clinical reliability. The complexity of the synthesis process stems from the large difference in the chemical properties of organic and inorganic components, which requires precise control of reaction conditions and process parameters, which increases the technical difficulty and cost input. The interfacial compatibility problem is due to the different surface energy and chemical bonding modes of the organic phase and the inorganic phase, which makes it difficult to form a stable and uniform combination and then affecting the material's overall performance.

At present, only one hybrid NCs has been approved. Fyarro is an organic-inorganic combination of sirolimus albumin-bound nanoparticle suspensions, which improves the water solubility, stability, and bioavailability of the drug [168]. In preclinical studies, *in vitro* experiments have shown a significant inhibitory effect on specific tumor cell lines. Animal model studies have shown that it can inhibit tumor growth without apparent toxic side effects, and can enrich tumor tissues to enhance targeting. In clinical trials, phase I established safety and tolerability, and phase II showed significant efficacy in malignant perivascular epithelioid cell tumors, with an overall response rate of 39 % and long-term response in some patients, although phase III trials are ongoing.

With the continuous progress of materials science and biotechnology, hybrid NCs are expected to make breakthroughs in the following two aspects: first, the development of more efficient and accurate synthesis methods to reduce the cost and improve the quality and performance stability of materials, such as the use of emerging nanotechnology and self-assembly processes to optimize the material preparation process [169]; The second is to deeply study the interaction mechanism of organic-inorganic interface and solve the interface compatibility problem through molecular design and surface modification, to create hybrid materials with better performance [170]. In summary, hybrid NCs have great potential in cancer therapy and other fields, but many obstacles exist in from laboratory to clinical. We need to overcome technical, economic and clinical validation problems, ensure safety and practicality, and avoid wasting resources to promote their development in medical treatment.

### 5.1 Core-shell structured nanocarriers

As the name suggests, the core-shell structure consists of a core and a shell of two or more parts. Multiple parts can be connected by covalent or other bond arrangements or by the complexation reaction between some substances and ions to form a shell that wraps around the core material, forming a composite structure [171]. The core-shell structure is usually designed for specific purposes to combine the advantages of different parts. Coating stable materials outside some unstable materials is a common method for drug delivery NCs. Core-shell structures can sometimes be used to improve the functionality of existing structural designs, such as introducing specific characteristics into nanosystems by constructing core-shell structures and adding various TME-responsive shells to NCs to achieve precise targeted therapy [172]. Core-shell materials have broad potential applications in drug delivery due to their diverse composition, structural flexibility, and physicochemical properties.

**Fig. 12:**
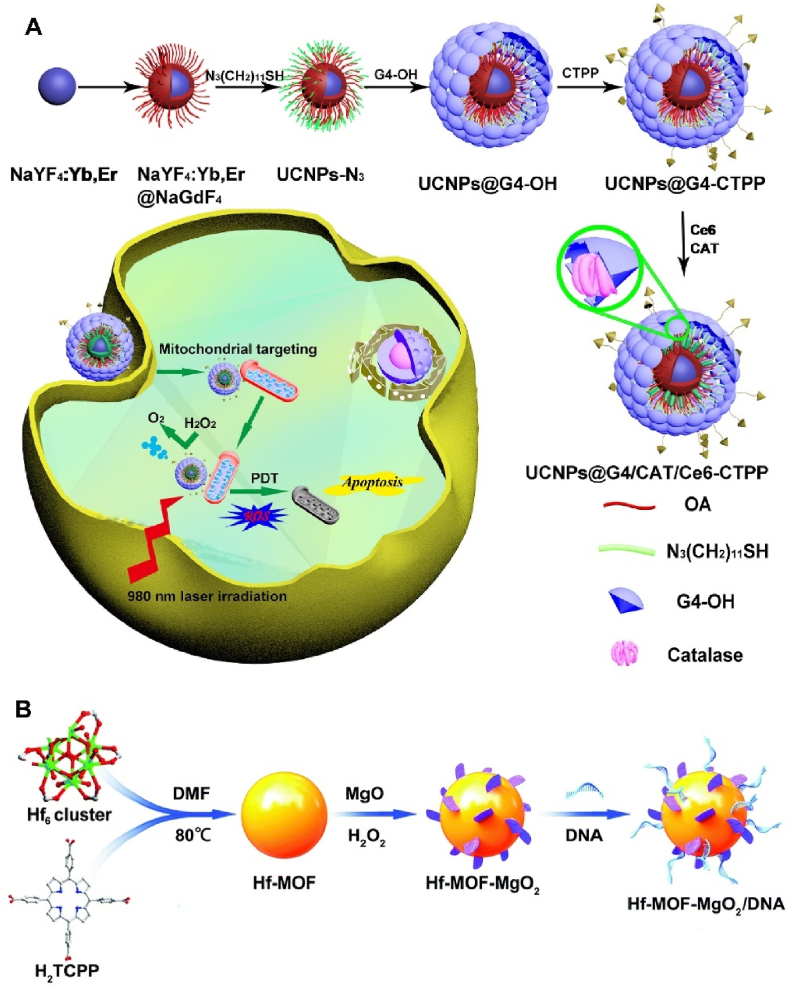
Targeted therapy of cancer with hybrid nanocarriers. (A) Schematic illustration for the synthesis of UCNPs@G4/Ce6/CAT-CTPP nanoparticles. Reproduced with permission [173]. Copyright 2020, Elsevier. (B) Schematic illustration of the synthesis and structure of Hf-MOF-MgO2/DNA. Reproduced with permission [174]. Copyright 2022, Royal Society of Chemistry.

Upconversion nanoparticles (UCNPs) can effectively create harmful ROS with limited cell selectivity by imposing event triggering at large tissue penetration depths. Selectivity is achieved by pointing a light beam at the intended target, and efficient triggering is achieved by converting near-infrared (NIR) light to visible light at the target locality. Liang et al. [173] constructed such an upconversion system using a unique thiolene-azide-acetylene click reaction chemistry. The original oleic acid ligand and dendrimer combined to create the multifunctional NCs UCNPs@G4/Ce6/CAT-CTPP (Fig. 12A). It is interesting to note that for catalytically enhanced PDT activated by NIR laser, "hydrophobic and hydrophilic pockets" built around a single upconversion nanoparticle are loaded with both the hydrophobic photosensitizer Chlorin e6 (Ce6) and the hydrophilic catalase enzyme (CTA). The mitochondrial targeting molecule (3-carboxypropyl) triphenyl phosphonium bromide (CTPP) was decorated outside the dendritic polymer to effectively target mitochondria. To combat tumor hypoxia, H_2_O_2_ is converted to H_2_O and O_2_ by catalase, and mitochondrial targeting significantly boosts PDT effectiveness. This work offers a new paradigm for constructing dendrimer shells with inorganic nanocrystal cores for drug delivery.

### 5.2 Metal-organic framework

Metal-organic frameworks (MOFs) are a promising new nanomaterial type to emerge in recent years. They represent a group of crystalline porous materials with periodic reticular skeletal structures created by coordinating metal ions (clusters) and organic ligands. The coordination of metal ions (clusters) and different organic ligands results in the formation of a family of different crystalline porous framework structures that serve various applications in gas separation and storage, energy catalysis, drug mitigation, and medical imaging. Applications of MOFs have benefitted from extensive research and rapid development. Sun et al. [174] reported that magnesium peroxide nanoparticles were coupled to an aptamer-functionalized metal-organic framework (Hf-MOF-MgO2/DNA) to target and improve PDT (Fig. 12B). Hf-MOF-MgO2/DNA created sufficient oxygen to address hypoxia concerns and improve PDT. The aptamer is modified for targeted therapy by linkage to ligands that precisely target tumor cells. The survival rate of 4T1 cells after PDT therapy *in vitro* was 15.15 %, while that of A549 cells was 28.69 % under the same conditions. This study demonstrates the generation of an effective ROS yield *in situ* creation and the efficient accumulation of PS at tumors, providing new opportunities for developing therapeutic nanomedicine.

## 6 Regulation of nanocarriers

When NCs are injected into the body, they encounter various physiological and biological obstacles that may affect their function and integrity. These obstacles include protein adsorption, diffusion, flow and shear, aggregation, phagocytic cell separation, and clearance of the kidneys, liver, and spleen [[175], [176], [177]]. By adjusting the size, shape, rigidity, and surface properties, NCs can overcome these biological obstacles, enrich their functions, and improve treatment efficiency [[178], [179], [180]]. However, many studies on such strategies still find it difficult to truly overcome these physiological barriers, mainly due to the different requirements for the physicochemical properties of NCs at different transportation stages [181]. Table 3 summarizes some methods for overcoming obstacles.

**Table 3:** Regulation of nanocarriers for overcoming physiological barriers in the last five years (examples and mechanism).

Type	Method	Result	Ref.
Surface control	Lay-by-lay modification	Improved targeting ability	[224]
	Altered surface charge	Positively charged nanoparticles have a stronger ability to target tumor-associated macrophages	[225]
	From negative charge to positive charge	Enhanced tumor accumulation and cell penetration	[226]
	Adjusting the level of surface positive charge	Nanoparticles with moderate positive surface charge (approximately 20 mV) can achieve efficient accumulation in tumors and lymph nodes while simultaneously targeting dendritic cells and tumor cells	[200]
	Biomimetic membrane coating	Improved stability and dispersibility Higher signal intensity and reduction of biotoxicity	[227]
	Cell membrane coating	To achieve homologous targeting, efficient drug delivery, immune evasion, and long circulation time.	[228]
Change in size	Size-charge dual changeable	Enhanced tumor penetration, tumor targeting, and penetration Enhanced activation and antitumor immunity To promote chemotherapy, endocytosis of drugs, and autophagy induction	[45]
Shape effect	Comparing spherical and rod-shaped nanocarriers	Tumor cells prefer endocytic rod-shaped nanocarriers.	[186]
	Comparing spherical and star-shaped nanocarriers	Nanostars and nanospheres have the same protein crown; Gold nanostars have faster diffusion speed, larger movement range, and faster rotational dynamics.	[182]
	Shaped like erythrocyte nanodisks	Rapid release of drugs under hypoxic conditions; Increase the permeability of the tumor; It is relatively stable in the high-oxygen region	[189]
Other properties	Tuning the elasticity	Improved delivery efficiency	[229]

### 6.1 Shape

The shape is a fundamental property of NCs and plays an important role in the drug delivery system. It can affect the circulation time, cellular uptake, targeting ability, and intracellular trafficking in the body [182]. Understanding the shape of geometry on NCs *in vivo* can help us design better drug delivery systems. The shape of the particle has been shown to greatly influence the function of the particle and plays a crucial role in cell phagocytosis of NCs. Current literature suggests that nanoparticle shape assists in crossing the gap at the vessel wall and in tumor penetration, retention, and internalization. Li et al. [183] developed a bionic rod-shaped polymeric micelle with a structure similar to bacterial pathogens. The rod shapes exhibit greater drug loading and slow drug release compared to spherical micelles that exhibit fast drug release. They also show a high internalization rate of tumor cells in a simulated acidic TME. However, these studies mostly remain at the level of *in vitro* or animal models, and the *in vivo* environment is complex and variable, which may greatly reduce the existing results in practical applications. For example, multiple biological barriers and complex interactions between biomolecules in the body may interfere with the shape and function of NCs, making it difficult to fully replicate their advantages *in vitro* experiments. Although there have been findings in shape-based tumor cell targeting research, there is still a gap between clinical applications. Relying solely on differences in cellular uptake to determine targeting strategies is too simplistic and ignores the heterogeneity of tumor cells. The response of tumor cells to the shape of NCs may vary among different subtypes of tumor cells and individual patients, and the characteristics of tumor cells may also change during disease development.

**Fig. 13:**
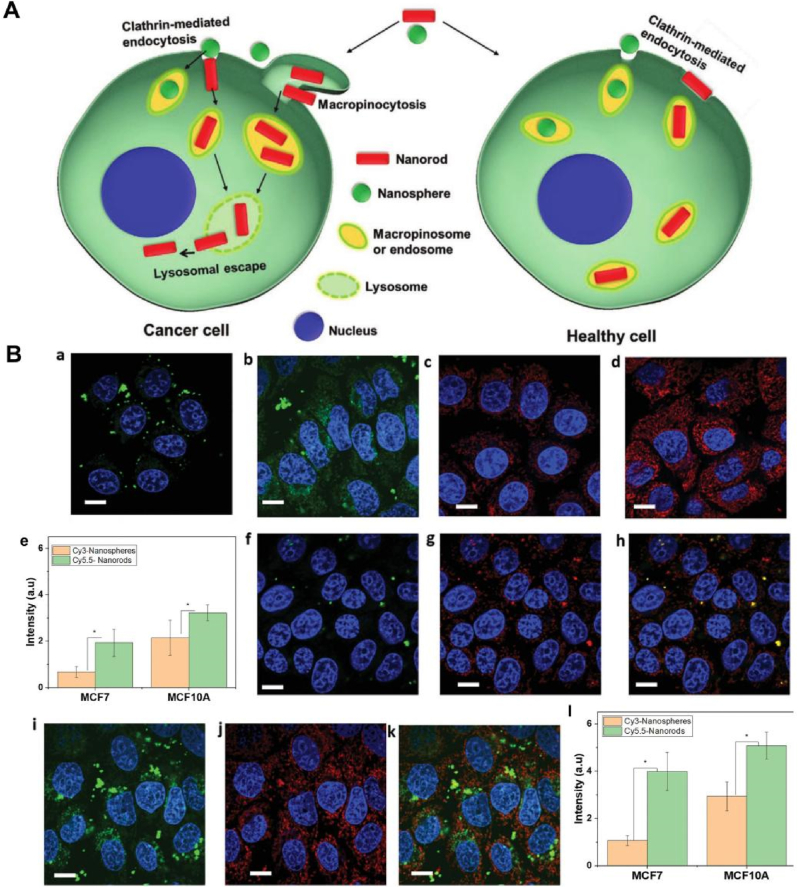
Effect of shape on nanocarriers. **(A**)Schematic illustration of the shape selection of nanoparticles on cancer and healthy cells. (B) The uptake of nanorods or nanospheres in MCF7 and MCF10A cells. (a) Uptake of nanospheres by MCF7 and (b) MCF10A cells. (c) Uptake of nanorods by MCF7 and (d) MCF10A cells. (e) Comparing the intracellular fluorescence intensity of nanorods and nanospheres in the two types of cells revealed that both had a greater uptake of nanorods. (f–h) Uptake of nanospheres (56 nm) and nanorod mixtures by MCF7 cells after 3.5 h. (i–k) Uptake of nanospheres and nanorod mixtures by MCF10A cells. Cy3 nanosphere channels (f, i). Cy5.5- Nanorod channels (g, j). Merge fluorescence images of two channels (h, k). (l) Comparison of intracellular fluorescence of a mixture of nanorods and nanospheres in two cell lines. (A–B) Reproduced with permission [186]. Copyright 2021, John Wiley and Sons.

Liu et al. [184] reported a spherical micelle that was transformed into nanofibres by radiation. The morphological transformation from spheres to fibers effectively increased the intra-tumor retention of nanodrugs. Decuzzi et al. and Geng et al. [69,185] found that rod-shaped nanoparticles were more fluid-like and dynamically unstable and sometimes failed to follow the predominant flow pattern as they propagated through the bloodstream. These hydrodynamic features increase the chances of the NCs crossing the gap in the vessel wall and point the way to identifying and fine-tuning the geometrical parameters that predominate over movement. Cong et al. [186] examined the endocytosis of two distinct nanoparticle morphologies (Fig. 13A and B) by comparing the take-up of nanospheres and nanorods in cancer and healthy cells. The cancer cells comprised pancreatic cancer cells (panc1 cells), pancreatic cancer cells (MCF7 cells), and breast epithelial cancer cells (MCF7). The healthy cells comprised normal breast epithelial cells (MCF10A) and non-tumorigenic, patient-derived cancer-associated fibroblasts. (CAFs). A combination of paired correlation microscopy and endocytosis inhibitor screening experiments was used to evaluate the endocytic pathway. While MCF10A cells mostly take up nanorods through clathrin-mediated endocytosis, MCF7 cells take them up through macro-pinocytosis and endocytosis. Cong et al. discovered, by comparing the endocytic behavior of cancer cells and healthy cells, that MCF7 cells take up more nanorods than MCF10A cells. Thus, MCF10A cells exhibit reduced nanorod metabolism and endocytosis. Pancreatic and breast cancer MCF7 cells can be targeted by nanorods, whereas healthy cells cannot. By modifying the structure of nanoparticles, this discovery offers the intriguing option of specifically targeting cancer cells instead of healthy ones.

However, there is currently a lack of in-depth understanding of how factors such as *in vivo* biological fluid dynamics (such as blood flow velocity and pressure changes) and tissue gap structure affect the shape and subsequent performance of NCs. Research on the influence of various factors in the tumor microenvironment on the shape of NCs is scattered and has not formed a systematic theory, making it difficult to guide clinical practice. We need to establish an *in vitro* model that is closer to physiological conditions and conduct real-time monitoring research *in vivo* to clarify the impact patterns, optimize the design of NCs, and ensure their functionality *in vivo*. Research on the mechanisms underlying the interactions between different tumor cell types, states, and NC shape is essential to achieving accurate tumor cell targeting based on NC shape. By establishing multiple tumor cell models and systematically exploring the relationship between the shape, surface properties, and tumor cell characteristics of NCs, combined with multimodal imaging technology, reliable targeted markers can be found to improve treatment efficacy and reduce side effects. Breaking through the limitations of existing technology is key, and it is necessary to develop more precise, efficient, and scalable NCs shape control technologies. We can explore new synthesis methods and manufacturing processes, introduce advanced techniques such as microfluidics [187] and self-assembly [188], and establish strict quality control standards to ensure shape consistency and stability, providing technical support for clinical applications. At the same time, it is necessary to establish a comprehensive and systematic security assessment system. Study the distribution, metabolism, excretion, and potential toxicity of NCs in the body from both short and long-term perspectives, with a focusing on safety issues related to shape changes such as harmful substance production, immune effects, and damage to normal tissues.

### 6.2 Size

**Fig. 14:**
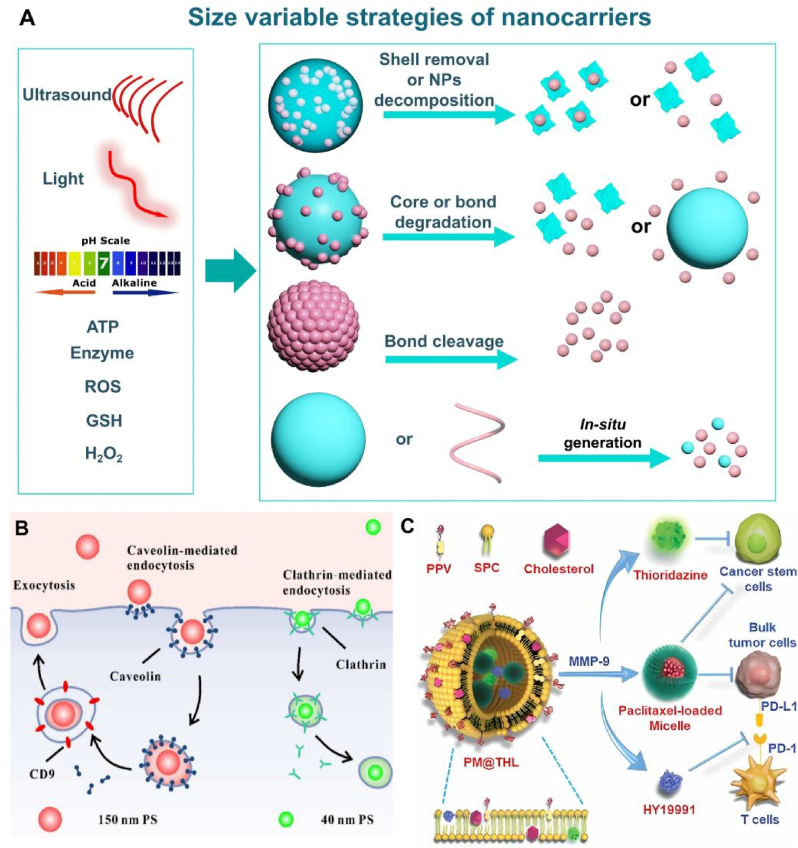
The effect of nanocarrier size on tumor targeted therapy. (A) Four size change strategies of nanocarriers induced by different stimulus responses. (B) Schematic illustration of different intracellular trafficking with two sizes of nanocarriers. Reproduced with permission [195]. Copyright 2017, American Chemical Society. (C) Structure and composition of PM@THL and treatment strategies. Reproduced with permission [58]. Copyright 2018, John Wiley and Sons.

Most drug delivery systems are administered through intravenous injection, which has high requirements for the particle size. Research has shown that nanoparticles with a particle size greater than 500 nm are prone to clearance by macrophages [189]. When the particle size ranges from 8 to 200 nm, it can effectively reduce clearance by the kidneys, liver, and spleen and increase internal circulation time. Nanoparticles with a diameter of less than 6–8 nm can be quickly expelled from the body through the kidneys [190]. In terms of targeted tumor therapy, we hope that NCs can circulate in the body for a long time to better rely on the EPR effect to passively target tumors, which requires a larger particle size of nanocomposites to achieve. However, the anti-tumor effect of chemotherapy drugs largely depends on their accumulation and permeation in the tumor, and large particle-size NCs are difficult to effectively penetrate tumor tissue. We hope NCs can become smaller particles when they reach the tumor site to penetrate deep into the tumor and effectively kill tumor cells. Given such contradictory issues, researchers have set out to study a class of variable-sized nano-drug delivery systems that can exist in larger particles during internal circulation, easily relying on the EPR effect to aggregate within tumors. Once they reach the tumor site and receive endogenous stimuli from TME (hypoxic environment, low pH, high levels of reactive oxygen species (ROS), overexpression of cancer-related enzymes) or exogenous stimuli (light, ultrasound), they will transform into small particles, facilitating their diffusion into the deep tumor. Stimulating NCs through various exogenous or endogenous pathways is a common means of size regulation; Table 4 summarizes some NCs that utilize stimulus response to achieve the adjustment of size. In general, nano drug delivery systems with variable sizes can be roughly divided into four categories: (1) Core-shell structures that can be peeled off [191]; (2) Small particles are connected to the surface of large particles [192]; (3) Small particles aggregate into clusters [193]; (4) In situ production of small particles [194]. These variable-size strategies can help NCs circulate in the body for a long time while penetrating tumor tissue, efficiently killing tumor cells (Fig. 14A). However, these strategies face many challenges in practical applications. It is difficult to achieve precise particle size transformation of NCs in the body. Precise material design and complex preparation processes are required to ensure that NCs maintain a stable large particle size state in blood circulation while responding to stimuli in a timely and effective manner to transform into small particles at the tumor site. Moreover, there are differences in the tumor microenvironment between different tumor types and individuals, which may affect the responsiveness of NCs to stimuli, leading to inconsistent particle size regulation and affecting treatment efficacy.

**Table 4:** Size variable strategies of nanocarriers.

Stimuli	Size_max_ (nm)	Size_min_ (nm)	Efficacy	Ref.
pH response	92 ± 21	14 ± 3	(1) Overcoming destabilized factors in the blood (2) Crossing blood-brain barrier/blood-brain tumor barrier (3) Promoting penetration and tumor targeting	[230]
pH response	112 ± 4.0	34	Improve tumor penetration and targeting capabilities	[231]
Ultrasonic triggering	188.4 ± 14.5	112.5 ± 11.6	(1) Removing protein crown (2) leaking targeting ligand (3) Inducing extravasation (4) nanocarriers extravasate from blood vessels into the tumor periphery via the ultrasonic-induced	[97]
GSH response	237.9 ± 6.4	125.8 ± 2.9	(1) Improving colloidal stability (2) Improving tumor permeability (3) Enhance the effect of immunochemotherapy	[232]
Bacterial secretion irritation	125	3.1	(1) Promoting penetration and tumor targeting (2) Prolonging circulation time in the body (3) Improving biodegradability	[190]
H_2_S response	130∼150	15	(1) Increasing intracellular endocytosis (2) Reducing intracellular copper elimination	[233]
GSH and pH response	14000	10300	Promoting penetration and tumor targeting	[234]

Wang et al. [195] investigated the endocytosis and exocytosis of two identical polystyrene (PS) nanoparticles. Studies using siRNAs and selective inhibitory medications have demonstrated that the diameter of PS NCs impacts how well they are taken up. While the caveola-mediated endocytic pathway primarily absorbs PS NCs at 150 nm, PS NCs at 40 nm are primarily internalized through the clathrin-mediated endocytic pathway (Fig. 14B). The findings of this work provide fresh insights into the design of drug carriers that specifically target specific receptors on the cell membrane. It reveals the specifics of various receptor-mediated interactions between cells and NCs of various sizes. The bigger PS NCs are directed by exosomes and delivered to the cell membrane, indicating they are released extracellularly. Larger NCs are selectively exported extracellularly, suggesting that particle size may affect how long drug carriers are retained by cells. In contrast, the smaller NCs become internalized and accumulate inside the cell. These recent discoveries provide insight into how cells and NCs interact and aid in designing nanoparticle structures that increase selectivity and safety. This study provides an important theoretical basis for the design of NCs, clarifying the influence of particle size on cellular uptake pathways and drug carrier retention time in cells, which helps to design more selective and safe nanoparticle structures. However, this study only focuses on polystyrene nanoparticles, and the generalizability of its conclusions in other types of NCs needs further verification. Different materials and structures of NCs may have different cellular interaction mechanisms.

Metastatic breast cancer may be resistant to chemoimmunotherapy due to the presence of cancer stem cells (CSC). Similarly, controlling the particle size and drug release of drug carriers used for multi-drug combinations is a key issue affecting treatment efficacy. Here, a cocktail therapy is reported, in which chemotherapy targeting large tumor cells and CSC and immune checkpoint blockade therapy, are integrated into a drug delivery system (Fig. 14C) [58]. The chemotherapy drug paclitaxel (PTX), the anti-CSC drug thiadiazine (THZ), and the PD-1/PD-L1 inhibitor HY19991 (HY) were all incorporated into the enzyme/pH double sensitive nanoscale structure with dual micelle liposomes. When nanoparticles transfer from circulation to tumor tissue, the decrease in pH and tumor extracellular matrix metalloproteinases (MMPs) trigger outer layer dissociation, exposing therapeutic molecules and reducing particle size, which is beneficial for pharmacokinetics and cellular absorption while achieving sequential drug release when needed. The device exhibits a tumor inhibition rate of up to 93.45 %. Combining multiple therapeutic molecules for transportation is expected to address the multidrug resistance of cancer patients to chemotherapy. This s strategy utilizes large particle sizes to achieve stable circulation in the body and allows for targeted sequential release of drugs, continuous elimination of tumors, and achieving ultra-high tumor inhibition ability. At the same time, it can recognize tumor T cells, effectively improving tumor invasion, metastasis, and other issues. However, this complex multifunctional nanocarrier faces many potential issues in the clinical translation process. Firstly, its preparation process is complex, involving integrating of multiple drugs and sensitive structures, making it difficult to achieve large-scale and standardized production, resulting in high costs and limiting its widespread application. Secondly, how to ensure the optimal synergistic effect of multiple drugs in the body while avoiding drug interactions that affect efficacy or increase toxicity is a problem that requires in-depth research.

### 6.3 Surface properties

**Fig. 15:**
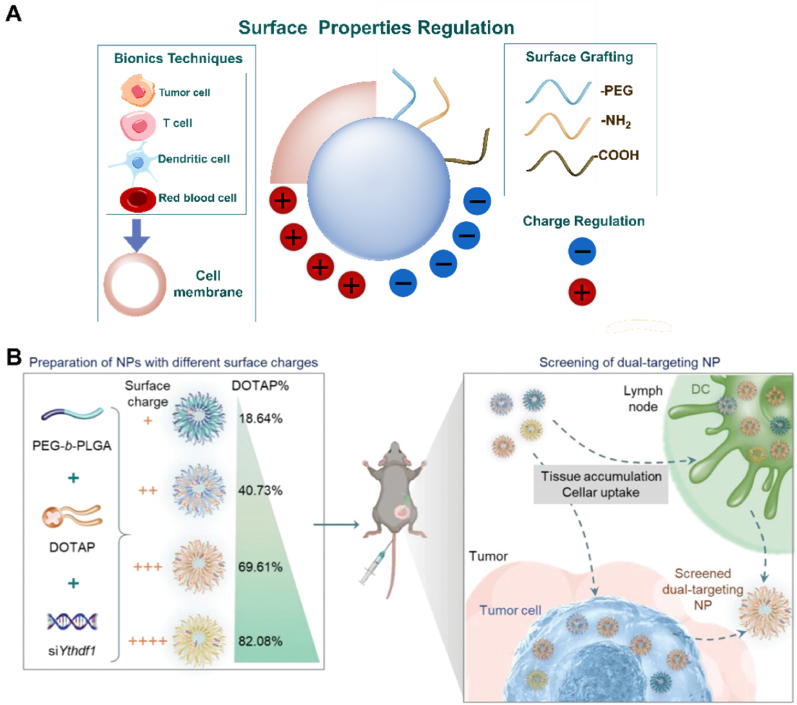
Schematic diagram of changing the physicochemical properties of nanocarriers through surface regulation. (A) Schematic diagram of changing the physicochemical properties of nanocarriers through biomimetic, grafting, and charge regulation methods. (B) NPs with different surface charges were prepared by adding varying amounts of cationic lipid 1,2-diole-3-trimethylpropane ammonium (DOTAP) to NPs. As shown in the figure, dual targeted nanomedicine can improve the tumor antigen presentation of dendritic cells (DCs) and tumor cells, thus enabling effective cancer immunotherapy. Inject NPs with different surface charges into tumor bearing mice. Observe the accumulation of NPs in tumors and lymph nodes and the internalization of NPs by DC and tumor cells. Select NPs that can be effectively internalized by DC and tumor cells as dual targeted NPs, while delivering drugs to both DC and tumor cells. Reproduced with permission [200]. Copyright 2023, John Wiley and Sons.

The physical and chemical properties, such as positive and negative electrophilicity and hydrophilicity/hydrophobicity on the surface of NCs, significantly impact on the biological properties of nanoparticles *in vitro* and *in vivo* [196]. These properties can affect the blood circulation of NCs, and their binding, distribution, and endocytosis ability with tumor cells. NCs systems face a significant challenge after administration, as they quickly adsorb large amounts of proteins, peptides, and other blood components upon entering the circulatory system. Adjusting the surface properties of NCs through biomimetic, grafting, and charge control methods can greatly improve the efficiency of targeted therapy (Fig. 15A) [197,198]. Research has shown that in blood circulation, neutral and negative surface potentials can reduce the adsorption of serum proteins on particle surfaces, assist in exposing ligands with targeting ability, achieve better precision treatment, and ensure long-term *in vivo* circulation. The modification of hydrophobic surfaces of nanoparticles can also solve the problem of protein adsorption. In biotechnology, a common strategy is to graft polyethylene glycol (PEG) onto the surface of NCs. Polyethylene glycol is a highly hydrophilic and flexible long-chain molecule that enhances the water solubility and stability of the payload and provides a steric barrier for the adsorption of serum proteins. It can also improve the dispersibility of some viscous carriers, such as nonminerals. Doxil® is a polyethylene glycol-modified liposome doxorubicin. Clinical studies have shown that Doxil can extend the *in vivo* clearance half-life of doxorubicin chemotherapy drugs to around 45 h. But PEG modification is not perfect either. Long-term use of PEG-modified NCs may trigger an immune response, leading to the producing anti-PEG antibodies in the body, affecting their long-term effectiveness and safety. In addition, PEG modification may interfere with the specific interaction between NCs and tumor cells. Although it can provide some steric hindrance to reduce non-specific adsorption, it may also hinder the binding of targeted ligands to tumor cell receptors to some extent, reducing targeting efficiency. Biomimetic technology has also become a commonly used means of improving the physical and chemical properties of NCs. Encapsulating NCs in cell membranes can enhance their affinity for cells and improve biocompatibility. At present, the commonly used cells for extracting cell membranes include tumor cells, red blood cells, white blood cells, dendritic cells and T cells. Impaired antigen presentation in dendritic cells (DCs) or tumor cells can hinder the effectiveness of cancer treatment. Here, Li et al. proposed a strategy of using dual-targeted nanomedicines to simultaneously improve the presentation of tumor antigens by dendritic cells and tumor cells. They regulate the surface charge of nanoparticles (NPs) by incorporating varying amounts of cationic lipids, thereby altering the accumulation of NP tissue and cellular targeting distribution *in vivo* (Fig. 15B). The results indicate that nanoparticles with a positive surface charge of about 20 mV have the best accumulation effect in tumors and lymph nodes. Dual targeted NP delivery of siRNA targeting YTH N6-methyladenosine RNA binding protein 1 (YTHDF1) to inhibit excessive antigen degradation in DC and tumor cells. For DC, downregulation of YTHDF1 promotes cross-cross-presentation of tumor antigens and cross-initiation of antigen-specific T cells. It enhances the presentation of endogenous tumor antigens for tumor cells, thereby improving the recognition and killing of tumor cells by triggering antigen -specific T cells. Dual-targeted nanomedicine produces effective anti-tumor immunity. It is theoretically reasonable to improve the biological properties of NCs by adjusting their surface properties. However, there may be many challenges in practical operation. Although neutral and negative surface potentials are indicated to reduce protein adsorption, this effect may not be as substantial as expected for different types of tumors and complex internal environments. For example, the heterogeneity of the tumor microenvironment may lead to local environmental changes that affect the actual effect of surface potential. Moreover, relying solely on surface potential adjustment may not entirely solve all the problems of NCs *in vivo*, such as penetration and distribution deep in tumor tissue.

## 7 Conclusions

TME, whose vascular and cellular composition differs markedly from normal tissues, provides a foundation for the application of NCs. NCs can take advantage of the characteristics of the TME to achieve precise drug delivery, for example, through passive or active targeting strategies. Each drug - delivery route has its own advantages and disadvantages. Oral administration is associated with the merit of convenience but is also accompanied by the drawback of low bioavailability. Intravenous injection can deliver drugs quickly; however, it entails several drawbacks, including causing pain to the patient, increasing the risk of infection, and being easily cleared. Moreover, individual differences in physiological and genetic factors also affect the therapeutic efficacy. Intrathecal administration can increase the local concentration of the drug, yet it is difficult to manage inflammatory reactions. The targeting strategies are divided into passive and active ones. Among these, passive targeting relies on the enhanced EPR effect. Although some studies have shown that the efficiency of passive targeting is limited and the mechanism of the EPR effect is facing challenges, active targeting can further enhance the targeting ability of NCs. However, the selection of targeting ligands is complex and faces difficulties in clinical translation.

NCs can be classified into three types: organic, inorganic, and hybrid. Organic NCs are usually more biocompatible, but their production cost is higher, and the production process is more complicated. Inorganic NCs have specific structural features and physical effects that make them easy to modify and functionalize. Nevertheless, they often face serious problems with degradation *in vivo*. Hybrid NCs combine the advantages of both but are difficult to synthesize.

Regulating the shape, size, and surface properties of NCs can effectively control their transport in the circulatory system to reduce the physiological barriers during drug delivery, such as reducing protein adsorption and enhancing the binding capacity between NCs and tumor cells. Several studies have demonstrated that the shape and size of NCs can influence the pathway and amount of their endocytosis by tumor cells. Surface properties can affect the mobility of NCs *in vivo* and their affinity for tumor cells. The effects of these properties on the relationship between NCs and targeting efficiency merit further exploration by researchers to enhance the tumor therapeutic ability of NCs.

NCs exhibit the potential to solve the problem of traditional chemotherapeutic drugs, but there are many key challenges in translating research results to the clinic. Difficulties translating NCs to the clinic are mainly due to technical, industrial, and economic challenges. At the technical level, the lack of deep understanding of TME poses a challenge to accurately designing NCs, affecting targeting and therapeutic effects. Preclinical research models cannot fully simulate the human environment, such as individual differences between different patients, and experimental results deviate from the actual clinical situation; *in vivo* behavior is not ideal, such as easy to aggregate, adsorption of plasma proteins, destruction by the immune system recognition, and the targeting of the large differences between the *in vivo* and *in vitro*, and intracellular susceptibility to lysosomal degradation and some of the long-term risk of toxicity. Long-term toxicity risks, Large-scale industrial production is complex, involving multiple aspects and steps; traditional purifying methods are inefficient, and quality control challenges abound; raw material batch-to-batch variation has a significant impact, and there is a lack of uniform regulatory protocols. Economically, high R&D and production costs make the market price expensive, and limited consumer affordability and imbalanced market demand impede the clinical translation of NCs.

With the continuous advancement of technology and in-depth research, the future development of NCs shows a broad and positive outlook. In terms of technological innovation, it is anticipated that more refinement and intelligence in the design of NCs will be needed. For example, through in-depth research on the properties of nanomaterials, new NCs with better performance will be developed, enabling them to better adapt to complex *in vivo* environments and improve stability, biocompatibility, and targeting. In terms of drug delivery mechanisms, more precise controlled release is achieved, such as the concentration of specific enzymes and the changes in specific molecules within the cell, to ensure that the drug is released at the exact time and location, maximizing therapeutic efficacy and reducing side effects. Meanwhile, with the help of artificial intelligence and machine learning technologies, the performance of NCs can be predicted and optimized, and personalized NCs systems can be designed according to different diseases and individual patient differences. Multidisciplinary integration will be a key driver for the development of NCs. The close cooperation of multiple disciplines, such as biology, materials science, medicine, and engineering, will bring new breakthroughs in the research and development of NCs. Through the concerted efforts of multidisciplinary teams, we will solve the current technical, industrial, and economic problems faced by NCs in clinical translation and accelerate their progress from laboratory to clinical application. With the increasing application of nanotechnology in medicine, the government and relevant regulatory agencies will strengthen the regulation of NCs R&D, production, and application and formulate more stringent scientific rules and standards. This will ensure the quality, safety, and efficacy of NCs, enhance public confidence in NCs technology, and promote its rational application in the clinic. Although there are still many difficulties, through continuous scientific research investment and interdisciplinary cooperation, NCs will undoubtedly play an increasingly important role in diagnosing and treating cancer.

## CRediT authorship contribution statement

**Hongxia Cheng:** Writing – original draft, Conceptualization. **Juan Liao:** Writing – review & editing. **Yuhan Ma:** Conceptualization. **Muhammad Tariq Sarwar:** Writing – review & editing. **Huaming Yang:** Conceptualization

## Declaration of competing interest

The authors declare that they have no known competing financial interests or personal relationships that could have appeared to influence the work reported in this paper.

## Data Availability

No data was used for the research described in the article.

## References

[1] Gu W., Meng F., Haag R., Zhong Z. (2021). Actively targeted nanomedicines for precision cancer therapy: concept, construction, challenges and clinical translation. J. Contr. Release.

[2] Deng C., Zhuang R., Ying Z., Tu J., Xu X., Sun C., Jiang L. (2022). Non‐invasive transdermal delivery systems with deep tissue penetrating ability for local ROS‐modulating chemotherapy. Adv. Funct. Mater..

[3] Van Akkooi A.C.J., Schadendorf D., Eggermont A.M.M. (2023). Alternatives and reduced need for sentinel lymph node biopsy (SLNB) staging for melanoma. Eur. J. Cancer.

[4] Majumder J., Minko T. (2021). Multifunctional and stimuli-responsive nanocarriers for targeted therapeutic delivery. Expet Opin. Drug Deliv..

[5] Berardi R., Caramanti M., Savini A., Chiorrini S., Pierantoni C., Onofri A., Ballatore Z., De Lisa M., Mazzanti P., Cascinu S. (2013). State of the art for cardiotoxicity due to chemotherapy and to targeted therapies: a literature review. Crit. Rev. Oncol. Hematol..

[6] Xu Y., Guo Y., Zhang C., Zhan M., Jia L., Song S., Jiang C., Shen M., Shi X. (2022). Fibronectin-coated metal–phenolic networks for cooperative tumor chemo-/chemodynamic/immune therapy via enhanced ferroptosis-mediated immunogenic cell death. ACS Nano.

[7] Hughes K.A., Misra B., Maghareh M., Bobbala S. (2023). Use of stimulatory responsive soft nanoparticles for intracellular drug delivery. Nano Res..

[8] Torchilin V.P. (2014). Multifunctional, stimuli-sensitive nanoparticulate systems for drug delivery. Nat. Rev. Drug Discov..

[9] Dai Y., Yang Z., Cheng S., Wang Z., Zhang R., Zhu G., Wang Z., Yung B.C., Tian R., Jacobson O., Xu C., Ni Q., Song J., Sun X., Niu G., Chen X. (2018). Toxic reactive oxygen species enhanced synergistic combination therapy by self-assembled metal-phenolic network nanoparticles. Adv. Mater..

[10] Younis M.A., Tawfeek H.M., Abdellatif A.A.H., Abdel-Aleem J.A., Harashima H. (2022). Clinical translation of nanomedicines: challenges, opportunities, and keys. Adv. Drug Deliv. Rev..

[11] Chen H., Zhang W., Zhu G., Xie J., Chen X. (2017). Rethinking cancer nanotheranostics. Nat. Rev. Mater..

[12] Blanco E., Shen H., Ferrari M. (2015). Principles of nanoparticle design for overcoming biological barriers to drug delivery. Nat. Biotechnol..

[13] Kobayashi H., Watanabe R., Choyke P.L. (2013). Improving conventional enhanced permeability and retention (EPR) effects; what is the appropriate target?. Theranostics.

[14] Matsumura Y., Maeda H. (1986). A new concept for macromolecular therapeutics in cancer chemotherapy: mechanism of tumoritropic accumulation of proteins and the antitumor agent smancs. Cancer Res..

[15] Sun R., Xiang J., Zhou Q., Piao Y., Tang J., Shao S., Zhou Z., Bae Y.H., Shen Y. (2022). The tumor EPR effect for cancer drug delivery: current status, limitations, and alternatives. Adv. Drug Deliv. Rev..

[16] Peer D., Karp J.M., Hong S., Farokhzad O.C., Margalit R., Langer R. (2007). Nanocarriers as an emerging platform for cancer therapy. Nat. Nanotechnol..

[17] Vincent M.P., Navidzadeh J.O., Bobbala S., Scott E.A. (2022). Leveraging self-assembled nanobiomaterials for improved cancer immunotherapy. Cancer Cell.

[18] Bhattacharjee K., Prasad B.L.V. (2023). Surface functionalization of inorganic nanoparticles with ligands: a necessary step for their utility. Chem. Soc. Rev..

[19] Gao M., Yu F., Lv C., Choo J., Chen L. (2017). Fluorescent chemical probes for accurate tumor diagnosis and targeting therapy. Chem. Soc. Rev..

[20] Zheng T., Li G.G., Zhou F., Wu R., Zhu J.J., Wang H. (2016). Gold-nanosponge-based multistimuli-responsive drug vehicles for targeted chemo-photothermal therapy. Adv. Mater..

[21] Han J., Dong H., Zhu T., Wei Q., Wang Y., Wang Y., Lv Y., Mu H., Huang S., Zeng K., Xu J., Ding J. (2024). Biochemical hallmarks-targeting antineoplastic nanotherapeutics. Bioact. Mater..

[22] Park J., Choi Y., Chang H., Um W., Ryu J.H., Kwon I.C. (2019). Alliance with EPR effect: combined strategies to improve the EPR effect in the tumor microenvironment. Theranostics.

[23] Hadjidemetriou M., Kostarelos K. (2017). Nanomedicine: evolution of the nanoparticle corona. Nat. Nanotechnol..

[24] Dilliard S.A., Siegwart D.J. (2023). Passive, active and endogenous organ-targeted lipid and polymer nanoparticles for delivery of genetic drugs. Nat. Rev. Mater..

[25] Xu M., Song J. (2021). Targeted therapy in cardiovascular disease: a precision therapy era. Front. Pharmacol..

[26] Zhang Y., Wang Y., Li X., Nie D., Liu C., Gan Y. (2022). Ligand-modified nanocarriers for oral drug delivery: challenges, rational design, and applications. J. Contr. Release.

[27] Naeem S., Viswanathan G., Misran M.B. (2018). Liposomes as colloidal nanovehicles: on the road to success in intravenous drug delivery. Rev. Chem. Eng..

[28] Colby A.H., Kirsch J., Patwa A.N., Liu R., Hollister B., McCulloch W., Burdette J.E., Pearce C.J., Oberliels N.H., Colson Y.L., Liu K., Grinstaff M.W. (2023). Radiolabeled biodistribution of expansile nanoparticles: intraperitoneal administration results in tumor specific accumulation. ACS Nano.

[29] An S., Fu L. (2018). Small-molecule PROTACs: an emerging and promising approach for the development of targeted therapy drugs. EBioMedicine.

[30] Ma S., Kim J.H., Chen W., Li L., Lee J., Xue J., Liu Y., Chen G., Tang B., Tao W., Kim J.S. (2023). Cancer cell-specific fluorescent prodrug delivery platforms. Adv. Sci..

[31] Yang Y., Wu H., Liu B., Liu Z. (2021). Tumor microenvironment-responsive dynamic inorganic nanoassemblies for cancer imaging and treatment. Adv. Drug Deliv. Rev..

[32] Zhen W., Weichselbaum R.R., Lin W. (2023). Nanoparticle-mediated radiotherapy remodels the tumor microenvironment to enhance antitumor efficacy. Adv. Mater..

[33] Heldin C.H., Rubin K., Pietras K., Ostman A. (2004). High interstitial fluid pressure - an obstacle in cancer therapy. Nat. Rev. Cancer.

[34] Agostinis P., Berg K., Cengel K.A., Foster T.H., Girotti A.W., Gollnick S.O., Hahn S.M., Hamblin M.R., Juzeniene A., Kessel D., Korbelik M., Moan J., Mroz P., Nowis D., Piette J., Wilson B.C., Golab J. (2011). Photodynamic therapy of cancer: an update. CA Cancer J. Clin..

[35] Shi J., Kantoff P.W., Wooster R., Farokhzad O.C. (2017). Cancer nanomedicine: progress, challenges and opportunities. Nat. Rev. Cancer.

[36] Thakkar S., Sharma D., Kalia K., Tekade R.K. (2020). Tumor microenvironment targeted nanotherapeutics for cancer therapy and diagnosis: a review. Acta Biomater..

[37] Li X., Lovell J.F., Yoon J., Chen X. (2020). Clinical development and potential of photothermal and photodynamic therapies for cancer. Nat. Rev. Clin. Oncol..

[38] He Y., Fan X., Wu X., Hu T., Zhou F., Tan S., Chen B., Pan A., Liang S., Xu H. (2022). pH-Responsive size-shrinkable mesoporous silica-based nanocarriers for improving tumor penetration and therapeutic efficacy. Nanoscale.

[39] Zhou Q., Xiang J., Qiu N., Wang Y., Piao Y., Shao S., Tang J., Zhou Z., Shen Y. (2023). Tumor abnormality-oriented nanomedicine design. Chem. Rev..

[40] Ilie M.D., Vasiljevic A., Bertolino P., Raverot G. (2022). Biological and therapeutic implications of the tumor microenvironment in pituitary adenomas. Endocr. Rev..

[41] Guo J., Dai J., Peng X., Wang Q., Wang S., Lou X., Xia F., Zhao Z., Tang B.Z. (2021). 9,10-Phenanthrenequinone: a promising kernel to develop multifunctional antitumor systems for efficient type I photodynamic and photothermal synergistic therapy. ACS Nano.

[42] Takahashi S., Kagami Y., Hanaoka K., Terai T., Komatsu T., Ueno T., Uchiyama M., Koyama-Honda I., Mizushima N., Taguchi T., Arai H., Nagano T., Urano Y. (2018). Development of a series of practical fluorescent chemical tools to measure pH values in living samples. J. Am. Chem. Soc..

[43] Ilie M.D., Vasiljevic A., Bertolino P., Raverot G. (2023). Biological and therapeutic implications of the tumor microenvironment in pituitary adenomas. Endocr. Rev..

[44] Jin J., Yuan P., Yu W., Lin J., Xu A., Xu X., Lou J., Yu T., Qian C., Liu B., Song J., Li L., Piao Y., Xie T., Shen Y., Tao H., Tang J. (2022). Mitochondria-targeting polymer micelle of dichloroacetate induced pyroptosis to enhance osteosarcoma immunotherapy. ACS Nano.

[45] Chen H., Guo Q., Chu Y., Li C., Zhang Y., Liu P., Zhao Z., Wang Y., Luo Y., Zhou Z., Zhang T., Song H., Li X., Li C., Su B., You H., Sun T., Jiang C. (2022). Smart hypoxia-responsive transformable and charge-reversible nanoparticles for the deep penetration and tumor microenvironment modulation of pancreatic cancer. Biomaterials.

[46] Dos Santos A.M., Carvalho S.G., Meneguin A.B., Sabio R.M., Gremiao M.P.D., Chorilli M. (2021). Oral delivery of micro/nanoparticulate systems based on natural polysaccharides for intestinal diseases therapy: challenges, advances and future perspectives. J. Contr. Release.

[47] Wang Z.H., Liu J.M., Li C.Y., Wang D., Lv H., Lv S.W., Zhao N., Ma H., Wang S. (2019). Bacterial biofilm bioinspired persistent luminescence nanoparticles with gut-oriented drug delivery for colorectal cancer imaging and chemotherapy. ACS Appl. Mater. Interfaces.

[48] Lee K.J., Ko E.J., Park Y.Y., Park S.S., Ju E.J., Park J., Shin S.H., Suh Y.A., Hong S.M., Park I.J., Kim K.P., Hwang J.J., Jang S.J., Lee J.S., Song S.Y., Jeong S.Y., Choi E.K. (2020). A novel nanoparticle-based theranostic agent targeting LRP-1 enhances the efficacy of neoadjuvant radiotherapy in colorectal cancer. Biomaterials.

[49] Zeng L., Gowda B.H.J., Ahmed M.G., Abourehab M.A.S., Chen Z.S., Zhang C., Li J., Kesharwani P. (2023). Advancements in nanoparticle-based treatment approaches for skin cancer therapy. Mol. Cancer.

[50] Nel J., Elkhoury K., Velot E., Bianchi A., Acherar S., Francius G., Tamayol A., Grandemange S., Arab-Tehrany E. (2023). Functionalized liposomes for targeted breast cancer drug delivery. Bioact. Mater..

[51] Truong Hoang Q., Ravichandran V., Nguyen Cao T.G., Kang J.H., Ko Y.T., Lee T.I., Shim M.S. (2022). Piezoelectric Au-decorated ZnO nanorods: ultrasound-triggered generation of ROS for piezocatalytic cancer therapy. Chem. Eng. J..

[52] Wang J., Zhang L., Chen M., Gao S., Zhu L. (2015). Activatable ferritin nanocomplex for real-time monitoring of caspase-3 activation during photodynamic therapy. ACS Appl. Mater. Interfaces.

[53] Habibi M., Berger R.D., Calkins H. (2021). Radiofrequency ablation: technological trends, challenges, and opportunities. Europace.

[54] Peverelli E., Treppiedi D., Mangili F., Catalano R., Spada A., Mantovani G. (2021). Drug resistance in pituitary tumours: from cell membrane to intracellular signalling. Nat. Rev. Endocrinol..

[55] Liu H.W., Chen L., Xu C., Li Z., Zhang H., Zhang X.B., Tan W. (2018). Recent progresses in small-molecule enzymatic fluorescent probes for cancer imaging. Chem. Soc. Rev..

[56] Kleinendorst S.C., Oosterwijk E., Bussink J., Westdorp H., Konijnenberg M.W., Heskamp S. (2022). Combining targeted radionuclide therapy and immune checkpoint inhibition for cancer treatment. Clin. Cancer Res..

[57] Zhu W., Chao Y., Jin Q., Chen L., Shen J.J., Zhu J., Chai Y., Lu P., Yang N., Chen M., Yang Y., Chen Q., Liu Z. (2023). Oral delivery of therapeutic antibodies with a transmucosal polymeric carrier. ACS Nano.

[58] Lang T., Liu Y., Zheng Z., Ran W., Zhai Y., Yin Q., Zhang P., Li Y. (2019). Cocktail strategy based on spatio-temporally controlled nano device improves therapy of breast cancer. Adv. Mater..

[59] Ilie M.-D., Vasiljevic A., Bertolino P., Raverot G. (2023). Biological and therapeutic implications of the tumor microenvironment in pituitary adenomas. Endocr. Rev..

[60] Dufort S., Bianchi A., Henry M., Lux F., Le Duc G., Josserand V., Louis C., Perriat P., Cremillieux Y., Tillement O., Coll J.L. (2015). Nebulized gadolinium-based nanoparticles: a theranostic approach for lung tumor imaging and radiosensitization. Small.

[61] Wibroe P.P., Anselmo A.C., Nilsson P.H., Sarode A., Gupta V., Urbanics R., Szebeni J., Hunter A.C., Mitragotri S., Mollnes T.E., Moghimi S.M. (2017). Bypassing adverse injection reactions to nanoparticles through shape modification and attachment to erythrocytes. Nat. Nanotechnol..

[62] Tang L., Yang X., Yin Q., Cai K., Wang H., Chaudhury I., Yao C., Zhou Q., Kwon M., Hartman J.A., Dobrucki I.T., Dobrucki L.W., Borst L.B., Lezmi S., Helferich W.G., Ferguson A.L., Fan T.M., Cheng J. (2014). Investigating the optimal size of anticancer nanomedicine. Proc. Natl. Acad. Sci. U. S. A.

[63] Mitragotri S., Burke P.A., Langer R. (2014). Overcoming the challenges in administering biopharmaceuticals: formulation and delivery strategies. Nat. Rev. Drug Discov..

[64] Abdella S., Abid F., Youssef S.H., Kim S., Afinjuomo F., Malinga C., Song Y., Garg S. (2023). pH and its applications in targeted drug delivery, Drug Discov. Today Off..

[65] Fan W., Wei Q., Xiang J., Tang Y., Zhou Q., Geng Y., Liu Y., Sun R., Xu L., Wang G., Piao Y., Shao S., Zhou Z., Tang J., Xie T., Li Z., Shen Y. (2022). Mucus penetrating and cell-binding polyzwitterionic micelles as potent oral nanomedicine for cancer drug delivery. Adv. Mater..

[66] Wu J., Ning P., Gao R., Feng Q., Shen Y., Zhang Y., Li Y., Xu C., Qin Y., Plaza G.R., Bai Q., Fan X., Li Z., Han Y., Lesniak M.S., Fan H., Cheng Y. (2020). Programmable ROS-mediated cancer therapy via magneto-inductions. Adv. Sci..

[67] Li X., Pan Z.a., Xiang C., Yuan Y., Chen J., Qing G., Ma J., Liang X.-J., Wu Y., Guo W. (2021). Structure transformable nanoparticles for photoacoustic imaging-guided photothermal ablation of tumors via enzyme-induced multistage delivery. Chem. Eng. J..

[68] Zhang Q., Zhao R., Zhang Y., Zou X., Zhang C.-y. (2023). One-step self-assembly of quantum dot-based spherical nucleic acid nanostructure for accurate monitoring of long noncoding RNA MALAT1 in living cells and tissues. Chem. Eng. J..

[69] Geng Y., Dalhaimer P., Cai S., Tsai R., Tewari M., Minko T., Discher D.E. (2007). Shape effects of filaments versus spherical particles in flow and drug delivery. Nat. Nanotechnol..

[70] Gref R., Minamitake Y., Peracchia M.T., Trubetskoy V., Torchilin V., Langer R. (1994). Biodegradable long-circulating polymeric nanospheres. Science.

[71] Wang X., Zhang H., Chen X., Wu C., Ding K., Sun G., Luo Y., Xiang D. (2023). Overcoming tumor microenvironment obstacles: current approaches for boosting nanodrug delivery. Acta Biomater..

[72] Maeda H., Nakamura H., Fang J. (2013). The EPR effect for macromolecular drug delivery to solid tumors: improvement of tumor uptake, lowering of systemic toxicity, and distinct tumor imaging in vivo. Adv. Drug Deliv. Rev..

[73] Chen Z., Kankala R.K., Long L., Xie S., Chen A., Zou L. (2023). Current understanding of passive and active targeting nanomedicines to enhance tumor accumulation. Coord. Chem. Rev..

[74] Costa S.A., Mozhdehi D., Dzuricky M.J., Isaacs F.J., Brustad E.M., Chilkoti A. (2018). Active targeting of cancer cells by nanobody decorated polypeptide micelle with bio-orthogonally conjugated drug. Nano Lett..

[75] Tian H., Zhang T., Qin S., Huang Z., Zhou L., Shi J., Nice E.C., Xie N., Huang C., Shen Z. (2022). Enhancing the therapeutic efficacy of nanoparticles for cancer treatment using versatile targeted strategies. J. Hematol. Oncol..

[76] Danhier F. (2016). To exploit the tumor microenvironment: since the EPR effect fails in the clinic, what is the future of nanomedicine?. J. Contr. Release.

[77] Clemons T.D., Singh R., Sorolla A., Chaudhari N., Hubbard A., Iyer K.S. (2018). Distinction between active and passive targeting of nanoparticles dictate their overall therapeutic efficacy. Langmuir.

[78] Sindhwani S., Syed A.M., Ngai J., Kingston B.R., Maiorino L., Rothschild J., MacMillan P., Zhang Y., Rajesh N.U., Hoang T., Wu J.L.Y., Wilhelm S., Zilman A., Gadde S., Sulaiman A., Ouyang B., Lin Z., Wang L., Egeblad M., Chan W.C.W. (2020). The entry of nanoparticles into solid tumours. Nat. Mater..

[79] Phillips R. (2023). Nanobody targets TNF and IL-6 for additive efficacy. Nat. Rev. Rheumatol..

[80] Srinivasarao M., Low P.S. (2017). Ligand-targeted drug delivery. Chem. Rev..

[81] Eda S., Nasibullin I., Vong K., Kudo N., Yoshida M., Kurbangalieva A., Tanaka K. (2019). Biocompatibility and therapeutic potential of glycosylated albumin artificial metalloenzymes. Nat. Catal..

[82] Suárez-García S., Solórzano R., Alibés R., Busqué F., Novio F., Ruiz-Molina D. (2021). Antitumour activity of coordination polymer nanoparticles. Coord. Chem. Rev..

[83] Safra T., Muggia F., Jeffers S., Tsao-Wei D.D., Groshen S., Lyass O., Henderson R., Berry G., Gabizon A. (2000). Pegylated liposomal doxorubicin (doxil): reduced clinical cardiotoxicity in patients reaching or exceeding cumulative doses of 500 mg/m2. Ann. Oncol..

[84] Pastori G., Guolo F., Guardo D., Minetto P., Clavio M., Miglino M., Giannoni L., Coviello E., Ballerini F., Galaverna F., Kunkl A., Colombo N., Grasso R., Lemoli R.M., Gobbi M. (2015). Fludarabine, cytarabine, daunoxome plus dasatinib has high efficacy with an acceptable toxicity profile as either consolidation or salvage regimen in adult Philadelphia positive acute lymphoblastic leukemia patients. Blood.

[85] Glantz M.J., Chamberlain M.C., Batchelor T., Eric W., Cavalli F., Shapiro W.R. (2006). Interaction between route of intra-CSF chemotherapy administration and efficacy of therapy in patients wtih neoplastic meningitis. J. Clin. Oncol..

[86] Ciolli S., Leoni F., Casini C., Breschi C., Bosi A. (2006). Liposomal doxorubicin (Myocet®) enhance the efficacy of bortezomib, dexamethasone plus thalidomide in refractory myeloma. Blood.

[87] Advani A., Earl M., Douer D., Rytting M., Bleyer A. (2007). Toxicities of intravenous (IV) pegasparaginase (ONCASPAR®) in adults with acute lymphoblastic leukemia (ALL). Blood.

[88] Green M.R., Manikhas G.M., Orlov S., Afanasyev B., Makhson A.M., Bhar P., Hawkins M.J. (2006). Abraxane®, a novel Cremophor®-free, albumin-bound particle form of paclitaxel for the treatment of advanced non-small-cell lung cancer. Ann. Oncol..

[89] Borges G.S.M., Lima F.A., Carneiro G., Goulart G.A.C., Ferreira L.A.M. (2021). All-trans retinoic acid in anticancer therapy: how nanotechnology can enhance its efficacy and resolve its drawbacks. Expet Opin. Drug Deliv..

[90] Dhiman N., Sarvaiya J., Mohindroo P. (2022). A drift on liposomes to proliposomes: recent advances and promising approaches. J. Liposome Res..

[91] Chang W., Shen J., Liu Z., Chen Q. (2023). Application of organic nanocarriers for intraocular drug delivery. J. Zhejiang Univ..

[92] Bangham A.D., Standish M.M., Watkins J.C. (1965). Diffusion of univalent ions across the lamellae of swollen phospholipids. J. Mol. Biol..

[93] Dutt Y., Pandey R.P., Dutt M., Gupta A., Vibhuti A., Raj V.S., Chang C.-M., Priyadarshini A. (2023). Liposomes and phytosomes: nanocarrier systems and their applications for the delivery of phytoconstituents. Coord. Chem. Rev..

[94] Filipczak N., Pan J., Yalamarty S.S.K., Torchilin V.P. (2020). Recent advancements in liposome technology. Adv. Drug Deliv. Rev..

[95] Wang X., Zhong X., Li J., Liu Z., Cheng L. (2021). Inorganic nanomaterials with rapid clearance for biomedical applications. Chem. Soc. Rev..

[96] Gouveia M.G., Wesseler J.P., Ramaekers J., Weder C., Scholten P.B.V., Bruns N. (2023). Polymersome-based protein drug delivery - quo vadis?. Chem. Soc. Rev..

[97] Wang G., Jiang Y., Xu J., Shen J., Lin T., Chen J., Fei W., Qin Y., Zhou Z., Shen Y., Huang P. (2023). Unraveling the plasma protein corona by ultrasonic cavitation augments active-transporting of liposome in solid tumor. Adv. Mater..

[98] Huang D., Wang J., Song C., Zhao Y. (2023). Ultrasound-responsive matters for biomedical applications. Innovation.

[99] Latreille P.L., Rabanel J.M., Le Goas M., Salimi S., Arlt J., Patten S.A., Ramassamy C., Hildgen P., Martinez V.A., Banquy X. (2022). In situ characterization of the protein corona of nanoparticles in vitro and in vivo. Adv. Mater..

[100] Zhang W., Meckes B., Mirkin C.A. (2019). Spherical nucleic acids with tailored and active protein coronae. ACS Cent. Sci..

[101] Wang S., Zhang J., Zhou H., Lu Y.C., Jin X., Luo L., You J. (2023). The role of protein corona on nanodrugs for organ-targeting and its prospects of application. J. Contr. Release.

[102] Li Y., Yang H.Y., Thambi T., Park J.-H., Lee D.S. (2019). Charge-convertible polymers for improved tumor targeting and enhanced therapy. Biomaterials.

[103] Alkahtani M.E., Elbadawi M., Chapman C.A.R., Green R.A., Gaisford S., Orlu M., Basit A.W. (2023). Electroactive polymers for on-demand drug release. Adv. Healthcare Mater..

[104] Zarrintaj P., Seidi F., Youssefi Azarfam M., Khodadadi Yazdi M., Erfani A., Barani M., Chauhan N.P.S., Rabiee N., Kuang T., Kucinska-Lipka J., Saeb M.R., Mozafari M. (2023). Biopolymer-based composites for tissue engineering applications: a basis for future opportunities. Compos. Part B-eng..

[105] Tu Z., Zhu Y., Xiao Y., Chen J., Shannon S., Zhang F., Li Z., Zhou J., Hu H., Ho T.-C., Gao W., Shao D., Leong K.W. (2022). Scavenging tumor-derived small extracellular vesicles by functionalized 2D materials to inhibit tumor regrowth and metastasis following radiotherapy. Adv. Funct. Mater..

[106] Wang G., Zhou Z., Zhao Z., Li Q., Wu Y., Yan S., Shen Y., Huang P. (2020). Enzyme-triggered transcytosis of dendrimer–drug conjugate for deep penetration into pancreatic tumors. ACS Nano.

[107] Li H.J., Du J.Z., Liu J., Du X.J., Shen S., Zhu Y.H., Wang X., Ye X., Nie S., Wang J. (2016). Smart superstructures with ultrahigh pH-sensitivity for targeting acidic tumor microenvironment: instantaneous size switching and improved tumor penetration. ACS Nano.

[108] Ghezzi M., Pescina S., Padula C., Santi P., Del Favero E., Cantù L., Nicoli S. (2021). Polymeric micelles in drug delivery: an insight of the techniques for their characterization and assessment in biorelevant conditions. J. Contr. Release.

[109] Wang X., Zhang M., Li Y., Cong H., Yu B., Shen Y. (2023). Research status of dendrimer micelles in tumor therapy for drug delivery. Small.

[110] Liu X., Liu W., Lu J., Li Q., Han W. (2021). Hybrid micelles enhance tumour therapy by remodelling biodistribution and improving intracellular drug release. Biomater. Sci..

[111] Yang L., Guo C., Jia L., Liang X., Liu C., Liu H. (2010). Dual responsive copolymer micelles for drug controlled release. J. Colloid Interface Sci..

[112] Cui J., Wang X., Li J., Zhu A., Du Y., Zeng W., Guo Y., Di L., Wang R. (2023). Immune exosomes loading self-assembled nanomicelles traverse the blood–brain barrier for chemo-immunotherapy against glioblastoma. ACS Nano.

[113] Grauer O., Jaber M., Hess K., Weckesser M., Schwindt W., Maring S., Stummer W., Wölfer J. (2016). Rthp-22. Inflammatory response after modified nanotherm and radiotherapy of recurrent glioblastoma neuro-oncol.

[114] Rahimkhoei V., Alzaidy A.H., Abed M.J., Rashki S., Salavati-Niasari M. (2024). Advances in inorganic nanoparticles-based drug delivery in targeted breast cancer theranostics. Adv. Colloid Interface Sci..

[115] Iravani S., Varma R.S. (2020). Green synthesis, biomedical and biotechnological applications of carbon and graphene quantum dots. A review, Environ. Chem. Lett..

[116] Priyadarshi R., Ezati P., Rhim J.-W. (2022). Synthesis, properties and food packaging applications of sulfur quantum dots: a review. Environ. Chem. Lett..

[117] Walther B.K., Dinu C.Z., Guldi D.M., Sergeyev V.G., Creager S.E., Cooke J.P., Guiseppi-Elie A. (2020). Nanobiosensing with graphene and carbon quantum dots: recent advances. Mater. Today.

[118] Li G., Liu Z., Gao W., Tang B. (2023). Recent advancement in graphene quantum dots based fluorescent sensor: design, construction and bio-medical applications. Coord. Chem. Rev..

[119] Chen H., Liu Z., Wei B., Huang J., You X., Zhang J., Yuan Z., Tang Z., Guo Z., Wu J. (2021). Redox responsive nanoparticle encapsulating black phosphorus quantum dots for cancer theranostics. Bioact. Mater..

[120] Liu Y., Zhang W., Zheng W. (2022). Surface chemistry of MXene quantum dots: virus mechanism-inspired mini-lab for catalysis. Chin. J. Catal..

[121] Qu S., Jia Q., Li Z., Wang Z., Shang L. (2022). Chiral NIR-II fluorescent Ag2S quantum dots with stereospecific biological interactions and tumor accumulation behaviors. Sci. Bull..

[122] Furey B.J., Stacy B.J., Shah T., Barba-Barba R.M., Carriles R., Bernal A., Mendoza B.S., Korgel B.A., Downer M.C. (2022). Two-photon excitation spectroscopy of silicon quantum dots and ramifications for bio-imaging. ACS Nano.

[123] Shen J., Zhu Y., Yang X., Li C. (2012). Graphene quantum dots: emergent nanolights for bioimaging, sensors, catalysis and photovoltaic devices. Chem. Commun..

[124] Li S., Su W., Wu H., Yuan T., Yuan C., Liu J., Deng G., Gao X., Chen Z., Bao Y., Yuan F., Zhou S., Tan H., Li Y., Li X., Fan L., Zhu J., Chen A.T., Liu F., Zhou Y., Li M., Zhai X., Zhou J. (2020). Targeted tumour theranostics in mice via carbon quantum dots structurally mimicking large amino acids. Nat. Biomed. Eng..

[125] Massaro M., Licandro E., Cauteruccio S., Lazzara G., Liotta L.F., Notarbartolo M., Raymo F.M., Sánchez-Espejo R., Viseras-Iborra C., Riela S. (2022). Nanocarrier based on halloysite and fluorescent probe for intracellular delivery of peptide nucleic acids. J. Colloid Interface Sci..

[126] Yin M., Tong J., Meng F., Liu C., Liu X., Fang F., He Z., Qin X., Liu C., Ni D., Gao Y., Liang H., Zhang X., Luo L. (2022). Near-infrared-II activatable symbiotic 2D carbon–clay nanohybrids for dual imaging-guided combinational cancer therapy. ACS Appl. Mater. Interfaces.

[127] Xie W., Chen Y., Yang H. (2023). Layered clay minerals in cancer therapy: recent progress and prospects. Small.

[128] Peixoto D., Pereira I., Pereira-Silva M., Veiga F., Hamblin M.R., Lvov Y., Liu M., Paiva-Santos A.C. (2021). Emerging role of nanoclays in cancer research, diagnosis, and therapy. Coord. Chem. Rev..

[129] Pereira I., Saleh M., Nunes C., Reis S., Veiga F., Paiva-Santos A.C. (2021). Preclinical developments of natural-occurring halloysite clay nanotubes in cancer therapeutics. Adv. Colloid Interface Sci..

[130] Avet F., Scrivener K. (2018). Investigation of the calcined kaolinite content on the hydration of limestone calcined clay cement (LC3). Cement Concr. Res..

[131] Dousova B., Lhotka M., Filip J., Kolousek D. (2018). Removal of arsenate and antimonate by acid-treated Fe-rich clays. J. Hazard Mater..

[132] Ng S., Plank J. (2012). Interaction mechanisms between Na montmorillonite clay and MPEG-based polycarboxylate superplasticizers. Cement Concr. Res..

[133] Roth W.J., Sasaki T., Wolski K., Song Y., Tang D.-M., Ebina Y., Ma R., Grzybek J., Kałahurska K., Gil B., Mazur M., Zapotoczny S., Cejka J. (2020). Liquid dispersions of zeolite monolayers with high catalytic activity prepared by soft-chemical exfoliation. Sci. Adv..

[134] Zhou Y., LaChance A.M., Smith A.T., Cheng H., Liu Q., Sun L. (2019). Strategic design of clay-based multifunctional materials: from natural minerals to nanostructured membranes. Adv. Funct. Mater..

[135] Liao J., Wang H., Liu N., Yang H. (2023). Functionally modified halloysite nanotubes for personalized bioapplications. Adv. Colloid Interface Sci..

[136] Yu X., Zhang Y.-C., Yang X., Huang Z., Zhang T., Yang L., Meng W., Liu X., Gong P., Forni A., Zheng Z., Liu B., Zhang P., Cai L., Tang B.Z. (2022). Bonsai-inspired AIE nanohybrid photosensitizer based on vermiculite nanosheets for ferroptosis-assisted oxygen self-sufficient photodynamic cancer therapy. Nano Today.

[137] Nie Y., Chen W., Kang Y., Yuan X., Li Y., Zhou J., Tao W., Ji X. (2023). Two-dimensional porous vermiculite-based nanocatalysts for synergetic catalytic therapy. Biomaterials.

[138] Ma L., Huang H., Feng W., Chen L., Xia L., Yu Y., Wang J., Chen Y. (2022). 2D catalytic, chemodynamic, and ferroptotic vermiculite nanomedicine. Adv. Funct. Mater..

[139] Xu Q., Yang Y., Lu J., Lin Y., Feng S., Luo X., Di D., Wang S., Zhao Q. (2022). Recent trends of mesoporous silica-based nanoplatforms for nanodynamic therapies. Coord. Chem. Rev..

[140] Wagner J., Gößl D., Ustyanovska N., Xiong M., Hauser D., Zhuzhgova O., Hočevar S., Taskoparan B., Poller L., Datz S., Engelke H., Daali Y., Bein T., Bourquin C. (2021). Mesoporous silica nanoparticles as pH-responsive carrier for the immune-activating drug resiquimod enhance the local immune response in mice. ACS Nano.

[141] Cai Y., Deng T., Pan Y., Zink J.I. (2020). Use of ferritin capped mesoporous silica nanoparticles for redox and pH triggered drug release in vitro and in vivo. Adv. Funct. Mater..

[142] Lee H., Choi M., Kim H.-E., Jin M., Jeon W.-J., Jung M., Yoo H., Won J.-H., Na Y.-G., Lee J.-Y., Seong H., Lee H.-K., Cho C.-W. (2022). Mannosylated poly(acrylic acid)-coated mesoporous silica nanoparticles for anticancer therapy. J. Contr. Release.

[143] Liang Y., Wang S., Yao Y., Yu S., Li A., Wang Y., Song J., Huo Z. (2022). Degradable self-destructive redox-responsive system based on mesoporous organosilica nano-vehicles for smart delivery of fungicide. Nanomaterials.

[144] Gao Z., Hou Y., Zeng J., Chen L., Liu C., Yang W., Gao M. (2017). Tumor microenvironment-triggered aggregation of antiphagocytosis 99mTc-labeled Fe_3_O_4_ nanoprobes for enhanced tumor imaging *in vivo*. Adv. Mater..

[145] Li L., Fang C.J., Ryan J.C., Niemi E.C., Lebrón J.A., Björkman P.J., Arase H., Torti F.M., Torti S.V., Nakamura M.C., Seaman W.E. (2010). Binding and uptake of H-ferritin are mediated by human transferrin receptor-1. Proc. Natl. Acad. Sci. USA.

[146] Lv Z., He S., Wang Y., Zhu X. (2021). Noble metal nanomaterials for NIR‐triggered photothermal therapy in cancer. Adv. Healthcare Mater..

[147] Yan T., Su M., Wang Z., Zhang J. (2023). Second near‐infrared plasmonic nanomaterials for photoacoustic imaging and photothermal therapy. Small.

[148] Yu Z., Chan W.K., Zhang Y., Tan T.T.Y. (2021). Near-infrared-II activated inorganic photothermal nanomedicines. Biomaterials.

[149] Chen J., Gong M., Fan Y., Feng J., Han L., Xin H.L., Cao M., Zhang Q., Zhang D., Lei D., Yin Y. (2022). Collective plasmon coupling in gold nanoparticle clusters for highly efficient photothermal therapy. ACS Nano.

[150] Chen Q., Li Z., Yu J., Xie Q., Lu H., Deng Y., Chen J., Zhu W., Huo L., Zhang Y., Song W., Lan J., Cai J., Huang Z., Wang Z., Zhao H. (2022). Novel gold nanoparticles targeting somatostatin receptor subtype two with near-infrared light for neuroendocrine tumour therapy. Nano Res..

[151] Kim H., Baek Y., Ha T., Choi D., Lee W.J., Cho Y., Park J., Kim S., Doh J. (2023). Gold nanoparticle‐carrying T cells for the combined immuno‐photothermal therapy. Small.

[152] Philip A., Kumar A.R. (2022). The performance enhancement of surface plasmon resonance optical sensors using nanomaterials: a review. Coord. Chem. Rev..

[153] Zhang Y., Sun Y., Dong X., Wang Q.-S., Zhu D., Mei L., Yan H., Lv F. (2022). A platelet intelligent vehicle with navigation for cancer photothermal-chemotherapy. ACS Nano.

[154] Xie H., Zhang C., Li T., Hu L., Zhang J., Guo H., Liu Z., Peng D., Li Z., Wu W., Gao J., Bi Z., Wang J., Zhang P., Kwok R.T.K., Lam J.W.Y., Guo Z., Xi L., Li K., Tang B.Z. (2023). Fast delivery of multifunctional NIR‐II theranostic nanoaggregates enabled by the photoinduced thermoacoustic process. Adv. Sci..

[155] Higbee-Dempsey E.M., Amirshaghaghi A., Case M.J., Bouché M., Kim J., Cormode D.P., Tsourkas A. (2020). Biodegradable gold nanoclusters with improved excretion due to pH-triggered hydrophobic-to-hydrophilic transition. J. Am. Chem. Soc..

[156] Sinatra L., Bandolik J.J., Roatsch M., Sönnichsen M., Schoeder C.T., Hamacher A., Schöler A., Borkhardt A., Meiler J., Bhatia S., Kassack M.U., Hansen F.K. (2020). Hydroxamic acids immobilized on resins (HAIRs): synthesis of dual‐targeting HDAC inhibitors and HDAC degraders (PROTACs). Angew. Chem. Int. Ed..

[157] Fan Y., Zhou Y., Lu M., Si H., Li L., Tang B. (2021). Responsive dual-targeting exosome as a drug carrier for combination cancer immunotherapy. Research 2021.

[158] Wang X., Ye L., He W., Teng C., Sun S., Lu H., Li S., Lv L., Cao X., Yin H., Lv W., Xin H. (2022). In situ targeting nanoparticles-hydrogel hybrid system for combined chemo-immunotherapy of glioma. J. Contr. Release.

[159] Shipunova V.O., Komedchikova E.N., Kotelnikova P.A., Zelepukin I.V., Schulga A.A., Proshkina G.M., Shramova E.I., Kutscher H.L., Telegin G.B., Kabashin A.V., Prasad P.N., Deyev S.M. (2020). Dual regioselective targeting the same receptor in nanoparticle-mediated combination immuno/chemotherapy for enhanced image-guided cancer treatment. ACS Nano.

[160] Patel R.B., Hernandez R., Carlson P., Grudzinski J., Bates A.M., Jagodinsky J.C., Erbe A., Marsh I.R., Arthur I., Aluicio-Sarduy E., Sriramaneni R.N., Jin W.J., Massey C., Rakhmilevich A.L., Vail D., Engle J.W., Le T., Kim K., Bednarz B., Sondel P.M., Weichert J., Morris Z.S. (2021). Low-dose targeted radionuclide therapy renders immunologically cold tumors responsive to immune checkpoint blockade. Sci. Transl. Med..

[161] Wang Q., Tian Y., Liu L., Chen C., Zhang W., Wang L., Guo Q., Ding L., Fu H., Song H., Shi J., Duan Y. (2021). Precise targeting therapy of orthotopic gastric carcinoma by siRNA and chemotherapeutic drug codelivered in pH-sensitive nano platform. Adv. Healthcare Mater..

[162] Wang R., Kim K.-H., Yoo J., Li X., Kwon N., Jeon Y.-H., Shin S.-k., Han S.S., Lee D.-S., Yoon J. (2022). A nanostructured phthalocyanine/albumin supramolecular assembly for fluorescence turn-on imaging and photodynamic immunotherapy. ACS Nano.

[163] Breuer E., Hebeisen M., Schneider M.A., Roth L., Pauli C., Frischer-Ordu K., Eden J., Pache B., Steffen T., Hübner M., Villeneuve L., Kepenekian V., Passot G., Gertsch P., Gupta A., Glehen O., Lehmann K. (2021). Site of recurrence and survival after surgery for colorectal peritoneal metastasis. J. Natl. Cancer Inst..

[164] Wang Q., Liu P., Wen Y., Li K., Bi B., Li B.-b., Qiu M., Zhang S., Li Y., Li J., Chen H., Yin Y., Zeng L., Zhang C., He Y., Zhao J. (2023). Metal-enriched HSP90 nanoinhibitor overcomes heat resistance in hyperthermic intraperitoneal chemotherapy used for peritoneal metastases. Mol. Cancer.

[165] Mulens-Arias V., Nicolás-Boluda A., Pinto A., Balfourier A., Carn F., Silva A.K.A., Pocard M., Gazeau F. (2021). Tumor-selective immune-active mild hyperthermia associated with chemotherapy in colon peritoneal metastasis by photoactivation of fluorouracil–gold nanoparticle complexes. ACS Nano.

[166] Qiu J., Liu Y., Xia Y. (2021). Radiolabeling of gold nanocages for potential applications in tracking, diagnosis, and image‐guided therapy. Adv. Healthcare Mater..

[167] He T., Jiang C., He J., Zhang Y., He G., Wu J., Lin J., Zhou X., Huang P. (2021). Manganese-dioxide-coating-instructed plasmonic modulation of gold nanorods for activatable duplex-imaging-guided NIR-II photothermal-chemodynamic therapy. Adv. Mater..

[168] Mann J.E. (2022). Sirolimus protein-bound particles (Fyarro™). Oncol. Times.

[169] Nabiyan A., Muttathukattil A., Tomazic F., Pretzel D., Schubert U.S., Engel M., Schacher F.H. (2023). Self-assembly of core–shell hybrid nanoparticles by directional crystallization of grafted polymers. ACS Nano.

[170] Yu Y., Peng Y., Shen W.-T., Zhou Z., Kai M., Gao W., Zhang L. (2024). Hybrid cell membrane-coated nanoparticles for biomedical applications. Small Structures.

[171] Yang Y., Zeng Z., Almatrafi E., Huang D., Zhang C., Xiong W., Cheng M., Zhou C., Wang W., Song B., Tang X., Zeng G., Xiao R., Li Z. (2022). Core-shell structured nanoparticles for photodynamic therapy-based cancer treatment and related imaging. Coord. Chem. Rev..

[172] Shi Y., Zhang Y., Zhu L., Miao Y., Zhu Y., Yue B. (2023). Tailored drug delivery platforms: stimulus‐responsive core–shell structured nanocarriers. Adv. Healthcare Mater..

[173] Liang S., Sun C., Yang P., Ma P., Huang S., Cheng Z., Yu X., Lin J. (2020). Core-shell structured upconversion nanocrystal-dendrimer composite as a carrier for mitochondria targeting and catalase enhanced anti-cancer photodynamic therapy. Biomaterials.

[174] Sun X., Zhang G., Ding X., Liu Y., Chen K., Shi P., Zhang S. (2022). A DNA functionalized metal–organic framework combined with magnesium peroxide nanoparticles: targeted and enhanced photodynamic therapy. Mater. Chem. Front..

[175] Jiang K., Yu Y., Qiu W., Tian K., Guo Z., Qian J., Lu H., Zhan C. (2023). Protein corona on brain targeted nanocarriers: challenges and prospects. Adv. Drug Deliv. Rev..

[176] Bi Q., Song X., Zhao Y., Hu X., Yang H., Jin R., Nie Y. (2023). Mucus-penetrating nonviral gene vaccine processed in the epithelium for inducing advanced vaginal mucosal immune responses. Acta Pharm. Sin. B.

[177] Zhu Y., Song Y., Cao Z., Dong L., Lu Y., Yang X., Wang J. (2021). Magnetically actuated active deep tumor penetration of deformable large nanocarriers for enhanced cancer therapy. Adv. Funct. Mater..

[178] Li X., Jafari S.M., Zhou F., Hong H., Jia X., Mei X., Hou G., Yuan Y., Liu B., Chen S., Gong Y., Yan H., Chang R., Zhang J., Ren F., Li Y. (2023). The intracellular fate and transport mechanism of shape, size and rigidity varied nanocarriers for understanding their oral delivery efficiency. Biomaterials.

[179] Bao W., Tian F., Lyu C., Liu B., Li B., Zhang L., Liu X., Li F., Li D., Gao X., Wang S., Wei W., Shi X., Li Y. (2021). Experimental and theoretical explorations of nanocarriers' multistep delivery performance for rational design and anticancer prediction. Sci. Adv..

[180] Song Y.H., De R., Lee K.T. (2023). Emerging strategies to fabricate polymeric nanocarriers for enhanced drug delivery across blood-brain barrier: an overview. Adv. Colloid Interface Sci..

[181] Chen L., Zhao T., Zhao M., Wang W., Sun C., Liu L., Li Q., Zhang F., Zhao D., Li X. (2020). Size and charge dual-transformable mesoporous nanoassemblies for enhanced drug delivery and tumor penetration. Chem. Sci..

[182] Choo P., Liu T., Odom T.W. (2021). Nanoparticle shape determines dynamics of targeting nanoconstructs on cell membranes. J. Am. Chem. Soc..

[183] Li D., Tang Z., Gao Y., Sun H., Zhou S. (2016). A bio-inspired rod-shaped nanoplatform for strongly infecting tumor cells and enhancing the delivery efficiency of anticancer drugs. Adv. Funct. Mater..

[184] Liu R., Yu M., Yang X., Umeshappa C.S., Hu C., Yu W., Qin L., Huang Y., Gao H. (2019). Linear chimeric triblock molecules self‐assembled micelles with controllably transformable property to enhance tumor retention for chemo‐photodynamic therapy of breast cancer. Adv. Funct. Mater..

[185] Decuzzi P., Pasqualini R., Arap W., Ferrari M. (2009). Intravascular delivery of particulate systems: does geometry really matter?. Pharm. Res..

[186] Cong V.T., Wang W., Tilley R.D., Sharbeen G., Phillips P.A., Gaus K., Gooding J.J. (2021). Can the shape of nanoparticles enable the targeting to cancer cells over healthy cells?. Adv. Funct. Mater..

[187] Zhang Q., Kuang G., Wang L., Fan L., Zhao Y. (2024). Tailoring drug delivery systems by microfluidics for tumor therapy. Mater. Today.

[188] Kim J., Lee S., Kim Y., Choi M., Lee I., Kim E., Yoon C.G., Pu K., Kang H., Kim J.S. (2023). In situ self-assembly for cancer therapy and imaging. Nat. Rev. Mater..

[189] Zhang H., Zhang Y., Hu H., Yang W., Xia X., Lei L., Lin R., Li J., Li Y., Gao H. (2023). In situ tumor vaccine for lymph nodes delivery and cancer therapy based on small size nanoadjuvant. Small.

[190] Wei X., Mu D., Li Y., Zhao J., Zhou S. (2023). A tumor-associated bacteria-responsive magnetic exosome as a clearable T1 contrast agent for targeted imaging of tumors. Nano Today.

[191] He J., Zhang W., Zhou X., Xu F., Zou J., Zhang Q., Zhao Y., He H., Yang H., Liu J. (2023). Reactive oxygen species (ROS)-responsive size-reducible nanoassemblies for deeper atherosclerotic plaque penetration and enhanced macrophage-targeted drug delivery, Bioact. Mater.

[192] Wang M., Zhang L., Cai Y., Yang Y., Qiu L., Shen Y., Jin J., Zhou J., Chen J. (2020). Bioengineered human serum albumin fusion protein as target/enzyme/pH three-stage propulsive drug vehicle for tumor therapy. ACS Nano.

[193] Li F., Liang Z., Liu J., Sun J., Hu X., Zhao M., Liu J., Bai R., Kim D., Sun X., Hyeon T., Ling D. (2019). Dynamically reversible iron oxide nanoparticle assemblies for targeted amplification of T1-weighted magnetic resonance imaging of tumors. Nano Lett..

[194] Chen Y., Zhang X.-H., Cheng D.-B., Zhang Y., Liu Y., Ji L., Guo R., Chen H., Ren X.-K., Chen Z., Qiao Z.-Y., Wang H. (2020). Near-infrared laser-triggered in situ dimorphic transformation of BF_2_-azadipyrromethene nanoaggregates for enhanced solid tumor penetration. ACS Nano.

[195] Wang T., Wang L., Li X., Hu X., Han Y., Luo Y., Wang Z., Li Q., Aldalbahi A., Wang L., Song S., Fan C., Zhao Y., Wang M., Chen N. (2017). Size-dependent regulation of intracellular trafficking of polystyrene nanoparticle-based drug-delivery systems. ACS Appl. Mater. Interfaces.

[196] Zhao Z., Ukidve A., Krishnan V., Mitragotri S. (2019). Effect of physicochemical and surface properties on in vivo fate of drug nanocarriers. Adv. Drug Deliv. Rev..

[197] Guo Y., Ma Y., Chen X., Li M., Ma X., Cheng G., Xue C., Zuo Y.Y., Sun B. (2023). Mucus penetration of surface-engineered nanoparticles in various pH microenvironments. ACS Nano.

[198] Lei W., Yang C., Wu Y., Ru G., He X., Tong X., Wang S. (2022). Nanocarriers surface engineered with cell membranes for cancer targeted chemotherapy. J. Nanobiotechnol..

[199] Melero I., Castanon E., Alvarez M., Champiat S., Marabelle A. (2021). Intratumoural administration and tumour tissue targeting of cancer immunotherapies. Nat. Rev. Clin. Oncol..

[200] Li M., Zhou H., Fang M., Qu K., Wang Y. (2023). Cationic lipids-mediated dual-targeting of both dendritic cells and tumor cells for potent cancer immunotherapy. Adv. Funct. Mater..

[201] Barenholz Y. (2012). Doxil® — the first FDA-approved nano-drug: lessons learned. J. Contr. Release.

[202] Fassas A., Buffels R., Anagnostopoulos A., Gacos E., Vadikolia C., Haloudis P., Kaloyannidis P. (2002). Safety and early efficacy assessment of liposomal daunorubicin (DaunoXome) in adults with refractory or relapsed acute myeloblastic leukaemia: a phase I–II study. Br. J. Haematol..

[203] McClune B., Buadi F., Aslam N., Przepiorka D. (2005). Intrathecal liposomal cytarabine (depocyt) is safe and effective for prevention of meningeal disease in patients with acute lymphoblastic leukemia and high-grade lymphoma treated with the HyperCVAD regimen. Blood.

[204] F. Iuliano, S. Molica, A. Serra, P. Sterpone, A. Russo, D. Cilloni, A. Peta, Clinical and hematological improvement in patients receiving liposome doxorubicin (Myocet) therapy for myelofibrosis with myeloid metaplasia (MMM), J. Clin. Oncol. 24(18_suppl) 6593-6593.

[205] Singh A.P., Biswas A., Shukla A., Maiti P. (2019). Targeted therapy in chronic diseases using nanomaterial-based drug delivery vehicles. Signal Transduct. Targeted Ther..

[206] Sanchez E., Steinberg J.A., Li M., Chen H., Bonavida B., Deitcher S.R., Hagey A.E., Berenson J.R. (2008). In vivo anti-tumor efficacy of vincristine sulfate liposomes injection (VSLI, Marqibo®) in multiple myeloma. Blood.

[207] A.K. Larsen, C. Trindade, A. Bouygues, L.K. Louadj, S.K. Thouroude, S.G. Klinz, A. Kalra, J. Henriques, B. Chibaudel, A. De Gramont, A.E. Escargueil, P. Mesange, Influence of liposomal irinotecan (nal-IRI) and non-liposomal irinotecan, alone and in combination, on tumor growth and angiogenesis in colorectal cancer (CRC) models, J. Clin. Oncol. 36(4_suppl) 711-711.

[208] M. Donnette, G. Venton, L. Farnault, B. Pourroy, R. Costello, B. Lacarelle, L.H. Ouafik, J. Ciccolini, R. Fanciullino, Pharmacokinetics/pharmacodynamics of liposomal cytarabine (VYXEOS) in AML patients: influence of cytidine deaminase genetic polymorphisms, J. Clin. Oncol. 38(15_suppl) e19517-e19517.

[209] Germain M., Caputo F., Metcalfe S., Tosi G., Spring K., Åslund A.K.O., Pottier A., Schiffelers R., Ceccaldi A., Schmid R. (2020). Delivering the power of nanomedicine to patients today. J. Contr. Release.

[210] Linxweiler H., Thiesen J., Krämer I. (2024). Physicochemical stability of nab-paclitaxel (Pazenir) infusion dispersions in original glass vials and EVA infusion bags. Pharmaceutics.

[211] Adick A., Hoheisel W., Schneid S., Mulac D., Azhdari S., Langer K. (2023). Challenges of nanoparticle albumin bound (nab™) technology: comparative study of Abraxane® with a newly developed albumin-stabilized itraconazole nanosuspension. Eur. J. Pharm. Biopharm..

[212] Wang C., Wang H., Yang H., Xu C., Wang Q., Li Z., Zhang Z., Guan J., Yu X., Yang X., Yang X., Li Z. (2023). Targeting cancer-associated fibroblasts with hydroxyethyl starch nanomedicine boosts cancer therapy. Nano Res..

[213] Barkovich K.J., Zhao Z., Steinmetz N.F. (2023). iRGD-targeted physalis mottle virus like nanoparticles for targeted cancer delivery. Small Science.

[214] Xia Y., Gu M., Wang J., Zhang X., Shen T., Shi X., Yuan W.-E. (2024). Tumor microenvironment-activated, immunomodulatory nanosheets loaded with copper(II) and 5-FU for synergistic chemodynamic therapy and chemotherapy. J. Colloid Interface Sci..

[215] Mei Y., Qin X., Yang Z., Song S., Liu X., Wu C., Qian J., Huang X., Zhang Y., He W. (2024). Engineered a dual-targeting HA-TPP/A nanoparticle for combination therapy against KRAS-TP53 co-mutation in gastrointestinal cancers. Bioact. Mater..

[216] Li J., Lv Z., Guo Y., Fang J., Wang A., Feng Y., Zhang Y., Zhu J., Zhao Z., Cheng X., Shi H. (2023). Hafnium (Hf)-Chelating porphyrin-decorated gold nanosensitizers for enhanced radio–radiodynamic therapy of colon carcinoma. ACS Nano.

[217] Zhi S., Zhang X., Zhang J., Wang X.-y., Bi S. (2023). Functional nucleic acids-engineered bio-barcode nanoplatforms for targeted synergistic therapy of multidrug-resistant cancer. ACS Nano.

[218] Kang X., Zhang Y., Song J., Wang L., Li W., Qi J., Tang B.Z. (2023). A photo-triggered self-accelerated nanoplatform for multifunctional image-guided combination cancer immunotherapy. Nat. Commun..

[219] Juul C.A., Engel T.B., Fliedner F.P., Ringgaard L., Eliasen R., Melander F., Bak M., Kjær A., Henriksen J.R., Elema D.R., Hansen A.E., Andresen T.L. (2024). HER2-targeted, enzyme-activated liposomes show superior in vivo efficacy in an ovarian cancer model. J. Contr. Release.

[220] Ye J.-J., Yu W., Xie B.-R., Li K., Liu M.-D., Dong X., Chen Z.-X., Feng J., Zhang X.-Z. (2022). Self-reinforced cancer targeting (SRCT) depending on reciprocally enhancing feedback between targeting and therapy. ACS Nano.

[221] Piao C., Lee J., Kim G.E., Choe Y.H., Lee H., Hyun Y.-M. (2024). Targeted delivery of nanoparticle-conveyed neutrophils to the glioblastoma site for efficient therapy. ACS Appl. Mater. Interfaces.

[222] Wei Y., Chen Z., Huang C., Cheng H., Jiang X., Li S. (2024). A breast cancer targeted photodynamic degrader to activate immunotherapy through EGFR degradation mediated PD-L1 downregulation. Chem. Eng. J..

[223] Kim N., Kwon S., Kwon G., Song N., Jo H., Kim C., Park S., Lee D. (2024). Tumor-targeted and stimulus-responsive polymeric prodrug nanoparticles to enhance the anticancer therapeutic efficacy of doxorubicin. J. Contr. Release.

[224] Correa S., Boehnke N., Barberio A.E., Deiss-Yehiely E., Shi A., Oberlton B., Smith S.G., Zervantonakis I., Dreaden E.C., Hammond P.T. (2020). Tuning nanoparticle interactions with ovarian cancer through layer-by-layer modification of surface chemistry. ACS Nano.

[225] Deng H., Yang X., Wang H., Gao M., Zhang Y., Liu R., Xu H., Zhang W. (2024). Tailoring the surface charges of iron-crosslinked dextran nanogels towards improved tumor-associated macrophage targeting. Carbohydr. Polym..

[226] Jiang L., Zhou S., Zhang X., Li C., Ji S., Mao H., Jiang X. (2021). Mitochondrion-specific dendritic lipopeptide liposomes for targeted sub-cellular delivery. Nat. Commun..

[227] Srivastava I., Xue R., Jones J., Rhee H., Flatt K., Gruev V., Nie S. (2022). Biomimetic surface-enhanced Raman scattering nanoparticles with improved dispersibility, signal brightness, and tumor targeting functions. ACS Nano.

[228] Liu L., Pan D., Chen S., Martikainen M.V., Karlund A., Ke J., Pulkkinen H., Ruhanen H., Roponen M., Kakela R., Xu W., Wang J., Lehto V.P. (2022). Systematic design of cell membrane coating to improve tumor targeting of nanoparticles. Nat. Commun..

[229] Chen X., Zhang S., Li J., Huang X., Ye H., Qiao X., Xue Z., Yang W., Wang T. (2022). Influence of elasticity of hydrogel nanoparticles on their tumor delivery. Adv. Sci..

[230] Fang W., Song B., Han L., Tao J., Li Y., Tang R., Yan G. (2023). Spatiotemporal theranostic nanoprobes dynamically monitor targeted tumor therapy. Adv. Funct. Mater..

[231] Zang J., He R., Liu Y., Su R., Zhao Y., Zheng X., Liu Y., Chong G., Ruan S., Wang H., Xu D., Dong H., Li Y. (2022). A size/charge/targeting changeable nano-booster to realize synergistic photodynamic-immunotherapy with high safety. Chem. Eng. J..

[232] Zhang G., Zhan M., Zhang C., Wang Z., Sun H., Tao Y., Shi Q., He M., Wang H., Rodrigues J., Shen M., Shi X. (2023). Redox‐responsive dendrimer nanogels enable ultrasound‐enhanced chemoimmunotherapy of pancreatic cancer via endoplasmic reticulum stress amplification and macrophage polarization. Adv. Sci..

[233] Zhao F., Liang L., Wang H., Wang C., Su D., Ying Y., Li W., Li J., Zheng J., Qiao L., Mou X., Che S., Yu J. (2023). H_2_S‐Activated ion‐interference therapy: a novel tumor targeted therapy based on copper‐overload‐mediated cuproptosis and pyroptosis. Adv. Funct. Mater..

[234] Zeng X., Li P., Yan S., Liu B.-F. (2023). Reduction/pH-responsive disassemblable MOF-microbial nanohybrid for targeted tumor penetration and synergistic therapy. Chem. Eng. J..

